# Genome-Wide Patterns of Genetic Polymorphism and Signatures of Selection in *Plasmodium vivax*

**DOI:** 10.1093/gbe/evu267

**Published:** 2014-12-17

**Authors:** Omar E. Cornejo, David Fisher, Ananias A. Escalante

**Affiliations:** ^1^School of Biological Sciences, Washington State University; ^2^Center for Evolutionary Medicine and Informatics, the Biodesign Institute, Arizona State University; ^3^School of Life Sciences, Arizona State University; ^4^Present address: Institute for Genomics and Evolutionary Medicine, Temple University, Philadelphia, PA.

**Keywords:** *Plasmodium*, natural selection, genome variation, *Plasmodium vivax*, genome architecture

## Abstract

*Plasmodium vivax* is the most prevalent human malaria parasite outside of Africa. Yet, studies aimed to identify genes with signatures consistent with natural selection are rare. Here, we present a comparative analysis of the pattern of genetic variation of five sequenced isolates of *P. vivax* and its divergence with two closely related species, *Plasmodium cynomolgi* and *Plasmodium knowlesi*, using a set of orthologous genes. In contrast to *Plasmodium falciparum*, the parasite that causes the most lethal form of human malaria, we did not find significant constraints on the evolution of synonymous sites genome wide in *P. vivax*. The comparative analysis of polymorphism and divergence across loci allowed us to identify 87 genes with patterns consistent with positive selection, including genes involved in the “exportome” of *P. vivax*, which are potentially involved in evasion of the host immune system. Nevertheless, we have found a pattern of polymorphism genome wide that is consistent with a significant amount of constraint on the replacement changes and prevalent negative selection. Our analyses also show that silent polymorphism tends to be larger toward the ends of the chromosomes, where many genes involved in antigenicity are located, suggesting that natural selection acts not only by shaping the patterns of variation within the genes but it also affects genome organization.

## Introduction

Although rarely lethal, *Plasmodium vivax* is the most prevalent human malarial parasite outside of Africa (Gething et al. 2012). This parasitic disease has reemerged in many regions of the world that previously were considered to be malaria-free (Severini et al. 2004; Kim et al. 2009; Bitoh et al. 2011). There are several biological characteristics that make this parasite particularly resilient and a global health challenge (Mueller et al. 2009; Gething et al. 2012). One of those characteristics is that the *P. vivax* life cycle involves a dormant liver stage (hypnozoite) that can lead to relapses (Coatney et al. 1971). The radical cure of this stage requires the use of Primaquine, a drug that cannot be widely used in some areas due to complications when administrated to G6PD deficient patients (Bowman et al. 2004). An additional life history trait that makes the elimination of this parasite so difficult is that it appears to be efficiently transmitted at early stages of the infection when it may still be subclinical and, therefore, untreated (Boyd and Kitchen 1937; Bousema and Drakeley 2011).

Unfortunately, regardless of its high prevalence, there are still serious information gaps on its basic biology (Mueller et al. 2009). This situation is worsened by the lack of a long-term culture system and accessible animal models (Beeson and Crabb 2007; Panichakul et al. 2007). However, new approaches based on high-throughput technologies can facilitate the study of parasites collected from infected human populations, supporting the generation of full genomic information aimed at uncovering the genetic basis of *P. vivax* basic biological features (Acharya et al. 2011; Ray et al. 2012). Such approaches will benefit from a better understanding of the genome variation of this parasite. Indeed, *P. vivax* populations exhibit extraordinary genetic (Neafsey et al. 2012; Taylor et al. 2013) and phenotypic diversities that include differences in their periodicity patterns (periodic fevers associated with the release of parasites from red blood cells) and local adaptation to vectors (Joy et al. 2008; White 2011). One of the many factors that is likely maintaining the observed variation is natural selection.

Natural selection can generate patterns of variation that are detectable at the genomic level (Bustamante et al. 2005; Nielsen 2005; Pollard et al. 2006; Nielsen et al. 2007). However, as is the case in many complex processes, such genomic patterns are seldom the result of a single factor. Furthermore, processes broadly defined as population demography could mimic the effects of natural selection; those processes include parasite population expansions (e.g., epidemics), geographic structure, or dramatic reductions in population size (e.g., unstable transmission or massive treatment of the human population with effective drugs). Finally, patterns consistent with natural selection could be the result of processes that acted at different temporal and spatial scales. Regardless of these difficulties, genomic studies of selection provide valuable information about genes encoding for phenotypes of interest (e.g., drug resistance) or allow making inferences about selected phenotypes that were not originally considered (Escalante et al. 2004; Pollard et al. 2006; Nielsen et al. 2007).

There are several methods for studying natural selection, each of them with different underlying assumptions. They may detect different forms of natural selection, for example, purifying, directional, or balancing (Bustamante et al. 2005; Nielsen 2005; Pollard et al. 2006; Nielsen et al. 2007) or detect adaptive processes that took place at different time scales*.* One group of such tests relies on comparing samples of complete genomes from the species of interest with a different closely related species. In this context, the use of a closely related species allows for identifying either the ancestral and derived state of mutations in the species of interest or the relative rates of divergence and polymorphism in regions along the genome. Such methods are particularly useful when a priori information on the actual phenotypes associated with genetic changes is lacking (Escalante et al. 2004; Jeffares et al. 2007; Tetteh et al. 2009). Here, we apply such approaches when studying the genomic variation in *P. vivax.*

The origin of *P. vivax* as a species comprises a series of complex evolutionary events (Culleton et al. 2011; Taylor et al. 2013; Liu et al. 2014; Muehlenbein et al. 2014). However, the lineage leading to *P. vivax* in Homininae diverged from a monophyletic group of nonhuman primate malarial parasites that radiated in Southeast Asia (Escalante et al. 2005; Pacheco et al. 2012b). The macaque parasite *Plasmodium cynomolgi* seems to be the closest known species to *P. vivax* in Southeast Asia, and they share several biological characteristics (Coatney et al. 1971). Indeed, *P. cynomolgi* can occasionally infect humans (Ta et al. 2014). Considering that the extant hosts of these nonhuman primate parasites are mostly species of macaques that diverged from the lineage leading to humans at least 23.5 Ma (Glazko and Nei 2003), one can hypothesize that, in the process of adapting to a new and genetically distant host species, the ancient parasite populations were subjected to strong selective pressures imposed not only by a new vertebrate host but also by different species of vectors and other ecological variables. Identifying genes that were involved in such adaptations may provide new targets for interventions (Carlton et al. 2013).

In this study, we characterize the genome-wide distribution of the genetic variability within *P. vivax* (polymorphism) and its differentiation from *P. cynomolgi* (divergence) (Tachibana et al. 2012). We also explore the number of species-specific genes by comparing these two species with another nonhuman primate parasite *Plasmodium knowlesi* that is part of the same clade of nonhuman primate malarias from South East Asia (Escalante et al. 2005; Pacheco et al. 2012b). Even with a limited sample size, we identify numerous genes presenting signatures consistent with selection in *P. vivax*, including common antigens, as well as genes involved in the life cycle of the parasite. Our results provide new evidence showing that the distribution of the polymorphism in the *P. vivax* genome is not uniform but enriched toward genes located in the subtelomeric regions of the chromosomes, likely due to the effect of recombination rather than uneven mutation rates. Our results allow us to hypothesize that selection acts not only on genes but also on genomic architecture. We propose that evidence for this is the consequence of putative high recombination rates toward the subtelomeric regions and preferential distribution of genes putatively involved in antigenicity and host–parasite interaction toward these regions. The differential distribution of genes that are involved in the generation of antigenic variation toward these regions could make natural selection more efficient and thus not only genic variation but also genome architecture could result in adaptations in *P. vivax* and other malarial parasites.

## Materials and Methods

### Genomic Data

We obtained the sequences of protein coding genes from four newly published genomes of *P. vivax* (Neafsey et al. 2012, deposited in the GenBank under the IDs GCA_000320625.1, GCA_000320645.1, GCA_000320665.1, and GCA_000320685.1, those are also available at http://www.broadinstitute.org/annotation/genome/plasmodium_vivax/News.html, last accessed December, 2014). In addition, we used the genome obtained from the isolate Salvador I of *P. vivax* (Carlton et al. 2008) and the genomes of two parasites found in nonhuman primates that were used as outgroups: *P. cynomolgi* (Tachibana et al. 2012) and *P. knowlesi* (Pain et al. 2008). These genomes were obtained from PlasmoDB. All analyses involving the assessment and comparison of genetic diversity along chromosomes were done using the annotation of *P. vivax* Salvador I as a reference. Thus, such reference genome provided the genomic coordinates of the genes studied in this investigation, and synteny between isolates is assumed. Gene families are common in these species with variable number of paralogs (Carlton et al. 2008; Pain et al. 2008; Tachibana et al. 2012; Rice et al. 2014), those genes were excluded. We performed all analyses on a gene per gene basis including only those with clearly identifiable orthologous.

### Identification of Orthologous Proteins and Gene Alignments

For the identification of putative sets of orthologous proteins, we used OrthoMCL (Li et al. 2003). This program performs an all-versus-all BLAST search among the protein sequences from all seven genomes (five *P. vivax*, *P. cynomolgi,* and *P. knowlesi*). By so doing, this method identifies sets of coding genes with significant similarity. The resulting *P* values from the BLAST search are normalized and arranged in a similarity matrix that is then used to generate clusters of putative orthologous and/or recent paralogous proteins showing significant reciprocal similarity using a Markov Cluster algorithm (MCL). For the implementation of OrthoMCL, we specified an *E*-value cutoff of 10^−^^5^ and an inflation parameter of 1.5 for the MCL. Using this procedure, recent paralogs are identified as genes within the same genome that are reciprocally more similar to each other than any sequence from another genome. In this investigation, we excluded those putative paralogs and constrained the analyses to sets of unique orthologous genes. Thus, this study does not address the problem of selection acting on copy number variation in complex gene families. After pruning out paralogous clusters, we used the output of OrthoMCL to select and align sets of putative orthologous genes to estimate their genetic diversity within *P. vivax* and the divergence between the *Plasmodium* species considered. We aligned the amino acid-translated sequence of orthologous genes using MUSCLE then used it as a reference to align the nucleotide sequences. In this way, we ensured that the reading frames were maintained and gaps were included without artificial changes in the reading frame. Three different sets were defined: 1) orthologous clusters among the *P. vivax* genomes, used to assess the overall *P. vivax* genetic polymorphism; 2) orthologous clusters within the *P. vivax*–*P. cynomolgi* clade, which were used to perform neutrality tests that combine polymorphism and divergence data; and 3) an orthologous set with all three species, used to identify genes that were unique to each species.

In addition, we performed analyses to obtain the functional annotation of sets of unique orthologous genes found in *P. vivax* isolates with Blast2GO (Conesa et al. 2005; Conesa and Gotz 2008). For this, each set of unique orthologous genes for each isolate of *P. vivax* was compared with the Swissprot database to identify homologous sequences and then mapped to Gene Ontology (GO), Kyoto Encyclopedia of Genes and Genomes (KEGG), and InterPro annotation databases to retrieve GO terms and annotations. For each set, the proportion of annotated and nonannotated genes was calculated. We compared the number of genes for all GO categories in sets of unique genes present in each isolate of *P. vivax* against the number of genes for all GO categories present in the set of genes common to all isolates of *P. vivax*.

### Genetic Polymorphism and Divergence

Genetic diversity per locus was estimated from the number of segregating (variable) sites among orthologous sequences using the Watterson’s θ_S_ and the average pairwise nucleotide differences per site (π) per gene. Genetic divergence (*k*) between *P. vivax* isolates and *P. cynomolgi* was estimated using the average pairwise differences per gene. However, given the observed divergence between these two species (Escalante et al. 2005; Pacheco et al. 2012b), we corrected by the possibility of multiple mutations in the same site by using the Jukes–Cantor model. The Tajima’s *D* per locus was also estimated, allowing us to compare the standardized difference between the numbers of segregating (variant) sites per locus estimated under Watterson’s θ_S_ and the average pairwise differences π. The expected value for Tajima’s *D* is zero under the neutral model (single population under random mating and at equilibrium between mutation and genetic drift) since the statistics θ_S_ and π should have similar estimates. Although these *P. vivax* genomes are not part of a single population, the Tajima’s *D* distribution provides information on how the pattern of mutations changes across the genome. All these simple statistics were calculated in DNAsp v 4.5 (Rozas and Rozas 1995; Librado and Rozas 2009) and the libsequence software (Thornton 2003).

We investigated the distribution of genetic diversity in *P. vivax* along the chromosomes by exploring the relationship between genomic location and gene diversity (π) in fully orthologous genes using quantile regression methods on smoothing splines functions (Koenker and D'Orey 1987; Koenker et al. 1994; Koenker and Xiao 2002). By including only orthologous genes found in a 1:1:1:1:1 relationship among the *P. vivax* genomes in these analyses, we avoid that differentiation between paralogous copies could become a confounding factor in the analysis.

We used the genomic location of genes as described for the reference genome Salvador I (Carlton et al. 2008) and transformed the genetic location by estimating the distance from the middle of the chromosome to the end of the chromosome for each gene. We then defined ten internal breakpoints equally spaced along the chromosomes on which ordinary polynomials would be defined and created a design matrix (similar to what a multivariate linear regression linear matrix would look like) to perform quantile regression on each set of B-splines following Koenker, using the packages quantreg, MASS, and splines in R (Koenker and D'Orey 1987; Koenker et al. 1994; Koenker and Xiao 2002). Because some chromosomes also present an increase of diversity close to the center of the chromosome, and the analysis measures the changes of diversity as a function of distance, we masked all genes at a distance less than 100 thousand bp (1e5 bp) from the center for the analysis of all chromosomes. The reason for this was mainly statistical and aimed to minimize the effect of the intercept on the estimated values of the coefficients for the ten intervals. To test the significance of the estimated values describing the relationship between position and diversity in the genomes, we performed 1,000 randomizations of the gene location and performed the quantile regression analyses on B-splines for each randomized data set. This allowed creating an empirical null distribution for the coefficients. If a coefficient is larger than or smaller than 97.5% of the observations of the randomized distributions, we considered it to be significant. All statistical analyses on genetic diversity and divergence across loci and figures were executed in R v 2.15.3.

In addition to the description of genetic diversity and divergence, we estimated the effective number of codons (*N*_c_), which is a summary statistic that measures the bias in the use of synonymous codons (Wright 1990). Genes that are biased in their codon usage could potentially accumulate fewer synonymous substitutions. *N*_c_ estimates were carried out using the program ENCprime, which estimates the effective number of codons while taking into account the background nucleotide composition (Novembre 2002).

### Selection Analyses

To identify patterns consistent with natural selection acting across the *P. vivax* genome, we performed two different tests that use genome data: The modified version of Hudson–Kreitman–Augade test, so called two-dimensional HKA test, proposed by Innan (2006) and a generalized Bayesian version of the McDonald and Kreitman (MK) test (Eilertson et al. 2012). The modified version of the HKA test examines if the ratio of polymorphism (within *P. vivax*) to divergence (between *P. vivax* and *P. cynomolgi*) in a focal region (*r*_f_) is consistent with the average ratio of a set of reference loci considered to be neutral (*r*_ave_). As reference set of neutral loci, we selected 100 loci (see supplementary material and table S1, Supplementary Material online) that have orthologs in all *Plasmodium* species with no evidence of positive selection acting on them (determined by codon base phylogenetic methods and others, Pond et al. 2011) and that were not biased in terms of their codon usage in *P. vivax*. They are mainly housekeeping genes involved in metabolic pathways. These reference loci were used to generate a distribution for the ratio of polymorphism to divergence in the *P. vivax*–*P. cynomolgi* set. We then assessed if there were genes with a ratio *r*_f_ significantly smaller than that predicted given the reference panel indicating positive selection. For this, we used its implementation available at the authors’ website (http://www.sendou.soken.ac.jp/esb/innan/InnanLab/Index_En.html).

Like the HKA test, the original MK test uses the polymorphism and divergence to identify genes under selection (McDonald and Kreitman 1991). The genome-wide version of this test (SnIPRE, Eilertson et al. 2012) is formulated as generalized linear mixed model that explicitly incorporates the genome-wide effects as fixed effects and individual gene effects as random effects. This method estimates information jointly across loci, increasing the power to detect genes under selection, so it is especially advantageous where the number of genomes is low. A main genomic mutation parameter is estimated that describes the average number of synonymous changes genome wide, and then departures from this main effect at the gene level are explored in the framework of a log linear model. This framework allows us to assess the individual contribution of factors that explain the observed patterns of polymorphism versus divergence per gene per type of change (synonymous or nonsynonymous) and contrast those with the average genome wide. In this model, the counts of changes in the sequences are modeled as a Poisson variable and the two main effects that we are interested in are as follows:

- The effect of natural selection acting on a given gene. This is modeled by the sum of two terms: The fixed effect of the divergence between the species given that changes are nonsynonymous genome wide β^ND^ (average genome-wide selection effect) and the random effect of the divergence given that changes are nonsynonymous in a given gene β^NDG^ (gene-specific selection effect). The sum of these terms is a measure of the strength of selection per gene relative to neutrality. Such effect, if different than the genome wide average, could be negative or positive indicating negative or positive selection, respectively (Eilertson et al. 2012).
- The constraint in the accumulation of nonsynonymous polymorphic changes at a given gene. This is measured by the sum of β^N^ that models nonlethal nonsynonymous changes genome wide and β^NG^ that models nonlethal changes per gene. Thus, the sum β^N ^+ β^NG^ captures the variability on the accumulation of nonsynonymous changes per gene or the proportion of nonsynonymous polymorphic changes that are nonlethal in a given gene (Eilertson et al. 2012).

Although the effects of selection relate to the traditional way in which MK tables are interpreted (Eilertson et al. 2012), the effects of constraint are novel and are interpreted as the deviations on the proportion of nonsynonymous polymorphic changes on a gene compared with the genome-wide effects. Thus, a gene under strong constraint will show a lower relative proportion of replacement polymorphic changes when compared with the genome-wide average. This correlates to the proportion of unobserved lethal changes at a given gene (Eilertson et al. 2012). All these analyses were performed in R, with modified code provided by the authors (Eilertson et al. 2012). The effect of multiple testing was corrected by the Bonferroni correction and the less stringent FDR correction. All statistical tests and calculations were performed in the R statistical package v 2.15.3.

## Results

### Identification of Orthologous Genes

**Fig. 1.—:**
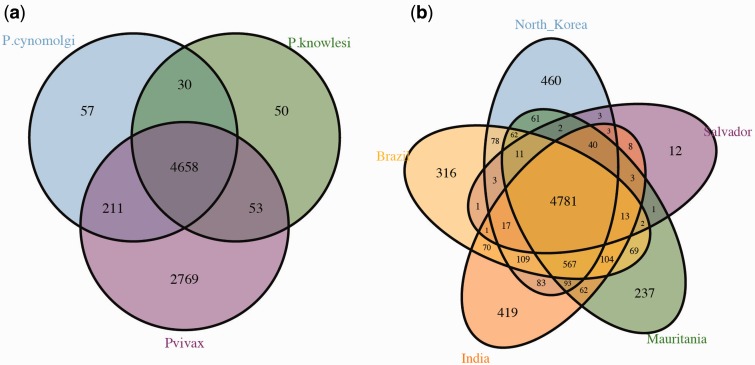
Determination of sets of orthologous genes. Venn diagrams showing the intersection of orthologous genes (*a*) shared by the three species considered in this study: *Plasmodium vivax*, *Plasmodium cynomolgi,* and *Plasmodium knowlesi* and (*b*) the number of orthologous genes shared by all five isolates of *P. vivax* considered in this study.

We identified 4,658 genes shared by all three species (after collapsing all clusters for which at least one *P. vivax* genome contains a gene represented in the set, fig. 1*a*), which is similar to the figure (4,613 genes) estimated in previous studies that have only considered the strain Salvador I of *P. vivax* for the comparison (Tachibana et al. 2012). However, the inclusion of four additional *P. vivax* genomes (Neafsey et al. 2012) yields a high number of unique genes for *P. vivax* (2,769 genes in this work vs. 204, see Tachibana et al. 2012) after intersecting with *P. cynomolgi* and *P. knowlesi* (fig. 1*a*). This estimate includes paralogs in gene families without clear orthologs in the three species or among all *P. vivax* isolates. When the analysis was restricted to the five *P. vivax* isolates (Carlton et al. 2008; Neafsey et al. 2012), we found that 4,781 genes are shared by all five isolates (fig. 1*b*). Furthermore, there were larger numbers of unique genes in the four newly sequenced isolates of *P. vivax*: North Korea (460 genes), Mauritania (237 genes), Brazil (316 genes), and India (419 genes), when compared with Salvador I (12 genes). Functional annotation analyses performed with Blast2GO suggest that only a few genes identified in each individual strain have known functions: 1) North Korea (7 of 460 genes, 1.5%), 2) Mauritania (5 of 237 genes 2%), 3) Brazil (4 of 316 genes, 1.3%), and 4) India (7 of 419 genes, 1.7%). This could be consistent with many of those genes being misannotated.

### Distribution of Genetic Diversity Genome Wide and Codon Usage Bias in *P. vivax*

**Fig. 2.—:**
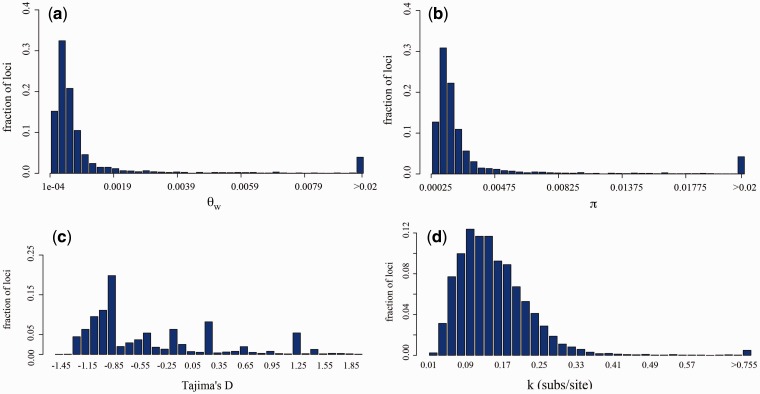
Distribution of polymorphism and divergence. Distributions of summary statistics per loci (fraction of loci) of genetic diversity estimated for *Plasmodium vivax*: (*a*) Watterson’s theta (θ_w_), which is a measure of the effective population size within the population and the mutation rate; (*b*) the average nucleotide pairwise difference between sequences (π); (*c*) Tajima’s *D*, which is a statistics that measures deviations from the neutral expectation under which θ_w_ and π are expected to be equal; and (*d*) the distribution of Jukes–Cantor corrected divergence between *P. vivax* and *Plasmodium cynomolgi*.

We estimated the polymorphism of *P. vivax* and the genetic divergences between *P. vivax* and each of the two nonhuman primate parasites, *P. cynomolgi* and *P. knowlesi,* on the 4,300 shared genes for which alignments were carefully curated, out of which only 2,492 genes have known products. Both distributions, for Watterson’s θ and π, showed a characteristic heavy tail (fig. 2*a* and *b*), and we reported the interquartile range (IQR) and the lower (*Q*_1_) and upper (*Q*_3_) quartiles of the distribution of each statistics estimated per locus. We found a relatively high-polymorphism rate in *P. vivax* (table 1) estimated from Watterson’s theta (${\bar{\theta }}_{\text{w}}$ = 4.78 × 10^−^^3^ mutations per site, IQR = 1.3 × 10^−^^3^, *Q*_1_ = 3.05 × 10^−^^4^, and *Q*_3_ = 1.57 × 10^−^^3^) and the average pairwise nucleotide differences ($\bar{\pi }$ =4.35 × 10^−^^3^, IQR = 1.21 × 10^−^^3^, *Q*_1_ = 2.67 × 10^−^^4^, and *Q*_3_ = 1.57 × 10^−^^3^). Overall, both measures of genetic diversity are similar genome wide, but when examined on a locus-by-locus basis we found a slight larger Watterson’s θ than π (see supplementary material and fig. S1, Supplementary Material online). This difference becomes evident when looking at the distribution of Tajima’s *D* statistics genome wide, which is slightly negative overall ($\bar{D}$ = −0.435, IQR = 0.90, *Q*_1_ =−0.973 and *Q*_3_ = −0.073, fig. 2*c*); although a considerable fraction of loci (18.7%) show positive Tajima’s *D* values, with 6.2% of loci presenting Tajima’s *D* values larger than 1. The relatively large variability of Tajima’s *D* is most likely due to the small sample size (*n* = 5) per locus, so most polymorphism appear in low frequency. Because of the potential low resolution of Tajima’s *D* in a locus-wise fashion with a limited sample, we estimated Tajima’s *D* in a sliding window analysis with 100-bp nonoverlapping windows and obtained similar results (mean *D* = −0.35, median *D* = −0.85, IQR = 1.06). The genetic divergence between *P. vivax* and *P. cynomolgi* is found to be around 14.5%, which is consistent with previous estimates, and its distribution is characterized by a heavy tail (${\bar{k}}_{\text{jc}}$ = 0.145 substitutions/site, IQR = 0.094, *Q*_1_ = 0.087 and *Q*_3_ = 0.181, fig. 2*d*). Meanwhile, the divergence between *P. vivax* and *P. knowlesi* is on average 18.3%, also with a distribution characterized by a heavy tail (${\bar{k}}_{\text{jc}}$ = 0.183 substitutions/site, IQR = 0.108, *Q*_1_ = 0.123 and *Q*_3_ = 0.231) (table 1).

**Fig. 3.—:**
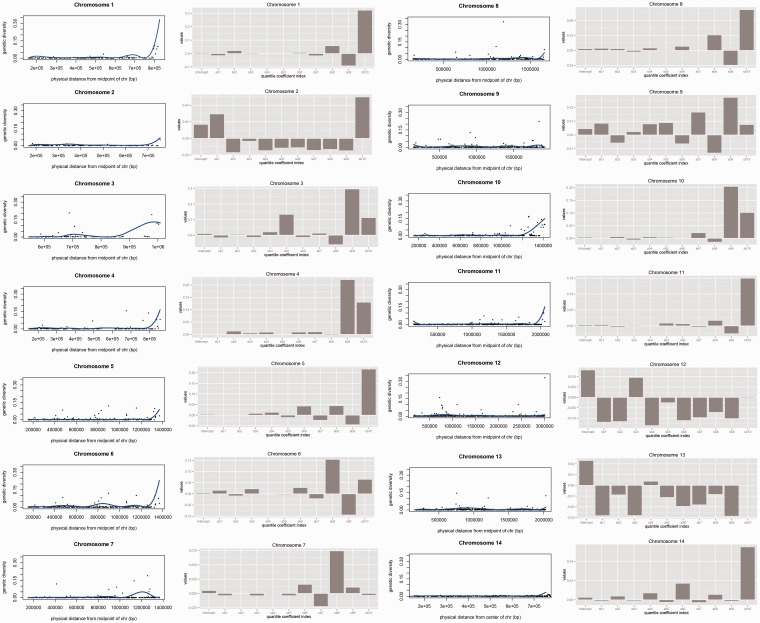
Distribution of synonymous polymorphism genome wide in *Plasmodium vivax* along each chromosome, from the center of the chromosomes (origin of X axis) to the subtelomeric regions (far right of X axis, on first and third panels). The blue lines are fitted B-splines that resulted from the best quantile regression fitting over the median of the genetic diversity as a function of their chromosomal position of the genes. It is noticeable the larger genetic polymorphism towards the end of chromosomes 1,2,3,4,5,8,9,10,11, and 14 of *P. vivax*. The regression coefficients for each one of the ten equally spaced intervals for the B-spline, as well as the intercept are shown as barplots in the second and fourth panels. Significance for each coefficient estimated from randomization tests is shown in the supplementary material.

Using the location of the genes annotated in the reference genome of *P. vivax* Salvador I (Carlton et al. 2008), we assessed the distribution of silent polymorphism along the genome (supplementary fig. S2, Supplementary Material online). There was a trend suggesting a larger genetic diversity, as estimated by silent polymorphism, in genes located toward telomeric regions (supplementary fig. S2, Supplementary Material online). We tested if this pattern was statistically significant by using quantile regression analyses on a B-spline design with ten equally spaced knots along chromosomal positions. For each one of the equally spaced knots, we estimated regression coefficients to explain genetic diversity as a function of distance from the center of the chromosome. It can be seen from the curves of the B-splines on the first and third panels of figure 3 and bar plots (on 2nd and 4th panels of fig. 3) showing the values of regression coefficients that genetic diversity is significantly larger toward the end of the chromosomes in 9 out of the 14 chromosomes of *P. vivax* (fig. 3). We tested the significance of these observations by bootstrap analysis of 1,000 pseudoreplicates in which we have randomized with replacement the gene location of each locus and repeated the analysis. We considered that coefficients of regression were significant if fitted values on the original data were significantly larger (or smaller) than 99% of the values estimated from the bootstrapped data sets. It can be seen that sb10 coefficients (the coefficients for the tenth knots) were larger than expected by chance for chromosomes 1, 2, 3, 4, 5, 6, 8, 10, 11, and 14 (see supplementary fig. S3, Supplementary Material online). These results are relevant given that many genes that could be implicated in *Plasmodium* antigenicity and virulence (vir genes and other gene families) are located toward the subtelomeric regions. A different and contrasting pattern was observed when examining the distribution of divergence genome wide, namely the distribution of differences between *P. vivax* and *P. cynomolgi*. The divergence was uniformly distributed along the genome (supplementary fig. S2, Supplementary Material online), with no clear increase toward the edges of the chromosomes. It is important to notice that genes with paralogous copies within and between all genomes (e.g., paralogs in the vir and msp-3 gene families) were excluded from this analysis, so the pattern of higher silent polymorphism toward the subtelomeric regions is both conservative and comparable with the divergent pattern with *P. cynomolgi*. Another pattern discovered using this analysis was that regions other than the subtelomeric exhibit significantly larger than expected polymorphism in chromosomes 1, 2, and 3, given the distribution of variation along the genome. Although those regions showing higher polymorphism in regions other than subtelomeres are significant, their absolute deviations from the average were smaller by comparison to those found toward the end of the chromosomes (fig. 3). We also found regions in the genome of *P. vivax* presenting lower diversity than expected.

**Fig. 4.—:**
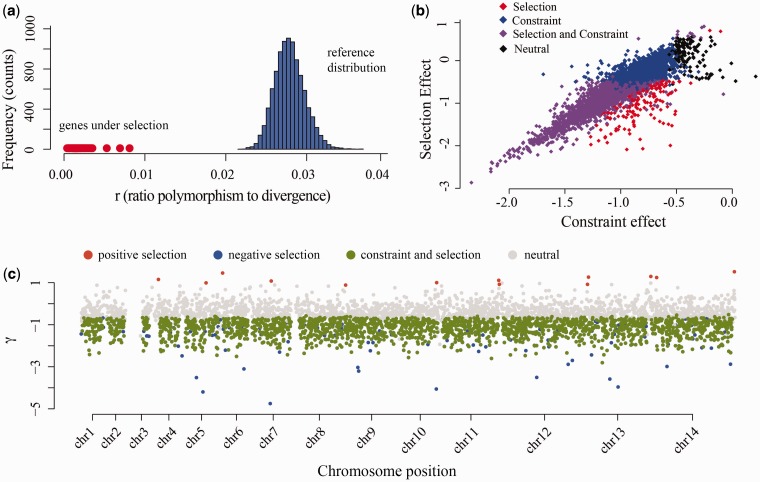
Identification of genes under selection. (*a*) Results of the modified HKA test for the identification of genes with decreased polymorphism to divergence ratio (*r*): in blue the reference distribution generated from 100 neutral loci is represented, and in red dots are represented the genes for which a significant difference has been identified. (*b*) General results from the Bayesian modified MK test (SnIPRE test): each diamond correspond to the inferred values of constraint (*x* axis, in the model) and the selection effect (*y* axis, $\beta^{\text{ND}}+\beta^{\text{NDG}}$ in the model) for each loci. Genes under positive selection are expected to have a significant positive selection effect (positive values in the *y* axis) and reduced constraint (larger values in *x* axis). There is a high prevalence of genes under strong negative selection and constraint (purple diamonds). All sites under selection (positive or negative) with no significant constraint are represented in red. (*c*) The distribution genome wide of the selection effects estimated per loci under SnIPRE. The red dots correspond to positively selected loci.

We analyzed the relative usage of synonymous codons along the genome in *P. vivax* by estimating the effective number of codons (*N*_c_) per gene per genome, using the modified statistic *N*_c_′ by (Novembre 2002), which takes into account biases in the nucleotide composition. We found that, on average, there is no significant codon bias in the *P. vivax* genomes (fig. 4, mean [*N*_c_′] = 52.18, *Q*_1_ = 49.68, *Q*_3_ = 54.30, IQR = 4.62); however, a reduced fraction of genes (∼4%, 170 genes) show evidence of codon bias as indicated by a relatively low *N*_c_ (<45), out of which 50% are identified as hypothetical proteins. In addition, the distribution of codon usage bias genome wide in *P. cynomolgi* was similar to that found in *P. vivax* (mean [*N*_c_′] = 53.75, *Q*_1_ = 52.03, *Q*_3_ = 56.22, IQR = 4.19), and there was a significant correlation between the codon usage in *P. vivax* genes and *P. cynomolgi* genes (Pearson’s *r* = 0.62, *P* value < 2.2e-16, see supplementary material and fig. S3, Supplementary Material online). Among the annotated protein coding genes presenting high codon usage bias in *P. vivax* (85 genes), around 50% were proteins involved in housekeeping processes (ribosomal proteins and other proteins involved in gene expression and protein synthesis, etc; see supplementary material and table S2, Supplementary Material online). Interestingly, 7% of the genes (six genes) correspond to members of the Pf/Pv–fam family (three Pv-fam-a, one Pv-fam-d, and two Phist Pf-fam-b genes), which are genes putatively responsible for antigenic response in *P. vivax* (Tachibana et al. 2012). Also, two genes implicated in gametocytes formation showed signatures of codon usage bias (a putative spermidine synthase, PVX_092065, and a putative Sperm-specific protein Don juan, PVX_085790). The average effective number of codons in *P. vivax* was much larger than that found in *Plasmodium falciparum* (*N*_c_ = 37.89), which is consistent with analyses performed on the strain Salvador I alone (Yadav and Swati 2012).

### Identification of Genes under Selection

The tests used, the modified HKA and the genomic MK tests, rely (in different ways) on the assumption that most of the genome will be accumulating variation following a neutral model (affected mainly by demographic effects), and this will allow us to identify genes that depart from the overall genome pattern as being under selection. For the analysis of genes using the HKA approach, we estimated the ratio of polymorphism to divergence to be between 0.01 and 0.03, in a subset of a 100 neutral genes from *P. vivax* that did not present a strong codon usage bias. The observed ratio of polymorphism to divergence was used to generate, via a rejection-sampling method, a posterior distribution of divergence time (*T*) between *P. vivax* and *P. cynomolgi* conditional on the observed ratio. This posterior distribution of divergence time *T* was then employed to estimate the null distribution of *r*_focal_ (fig. 4*a*, blue histogram) for each locus to test if *r*_f_ is consistent with the average *r*_f_ of the reference regions. After Bonferroni correction and manual examination of potential alignment issues, we found 79 genes presenting evidence of reduced ratio of polymorphism to divergence (1.83%, red dots in fig. 4*a*), out of which 41 have been identified as hypothetical proteins without known function and 38 have well-known annotated functions (supplementary material and table S3, Supplementary Material online).

Of the 38 annotated proteins with a signature consistent with positive selection, seven are part of different Pv-fam gene families, which are genes believed to play an important role in immune escape by *Plasmodium* from the host response to the infection (Kyes et al. 2007; Recker et al. 2011). Also, a DnaJ domain containing protein which could be part of the “exportome” repertoire of *P. vivax* was found to present signatures of positive selection (Sargeant et al. 2006). In addition to these proteins involved in the “exportome,” we find that a putative merozoite surface protein (MSP-7, PVX_082645) and a gene potentially expressed by *P. vivax* in the mosquito salivary glands (PVX_080550, small GTPase Rab1) also have signatures consistent with positive selection.

Some of the proteins identified as putatively under selection are not directly implicated in transcription or obviously interacting with the host immune response. For instance, we found three helicases (e.g., DEAD-BOX RNA helicase and PVX_117510) and a RNA-metabolizing metallo-beta-lactamase domain containing protein (PVX_095300) with signatures consistent with positive selection.

We analyzed the pattern of replacement and synonymous polymorphism and divergence with the generalized version of the MK. In a conventional MK, a Fisher exact test is used to identify genes under selection by identifying genes that depart from the expectation that $P_{\text{s}}/D_{\text{s}}\approx P_{\text{N}}/D_{\text{N}}$ , where *P*_S_ and *P*_N_ correspond to the counts of synonymous and nonsynonymous (replacement) polymorphic changes, and *D*_S_ and *D*_N_ correspond to the counts of the synonymous and nonsynonymous divergence changes. In the generalized linear version of the MK, we fit the counts of the changes to a model that tests whether the number of changes in a locus is significantly affected by the type of change: 1) nonsynonymous (*N*) and 2) fixed difference (*D*) or a combination of both (*ND*). Table 2 lists the effect terms and the significance for the fitting for the fixed and random effects of the terms of the model. We find evidence for a significant amount of mutational constraint (the sum of the genome wide effect of the polymorphism being a replacement change and the individual effect of a particular gene on the accumulation of replacement changes, resulting in a low proportion of nonlethal accumulation of replacement mutations) and negative selection (the individual effect of the reduced fixation of replacement changes in specific genes given the overall fixation rate of replacement changes) (fig. 4*b*). Genes colored in red, figure 4*b*, are those for which individual gene effects, β^ND^+ β^NDG^, are significantly different to the overall genome-wide effect, so they depart from the expectations under neutrality after controlling for the general effect of a mutation being nonsynonymous (β^N^+β^NG^). Only 13 of those genes were identified presenting significant evidence of positive selection (fig. 4*b* and *c*, see supplementary material and table S4, Supplementary Material online), those have a significantly larger contribution of fixed replacement differences and are potentially under lower constraint. Genes under purifying selection are those presenting a significantly negative β^ND^+β^NDG^ conditional on the proportion of replacement polymorphism, but they do not depart significantly from the fixed genome wide effect of having nonsynonymous changes (no detectable constraint to accumulate nonsynonymous changes). Genes depicted in purple are those showing effects consistent with purifying selection (given by β^ND^+ β^NDG^) that also show patterns indicating strong constraint (significant β^N^ + β^NG^). It is worth noting that the selection effect (positive or negative) is expected to identify patterns of natural selection acting on mildly deleterious mutations (negative selection) and advantageous mutations (positive selection), whereas a significant constraint effect in addition to negative selection allow identifying genes under strong negative or purifying selection (Eilertson et al. 2012).

**Table 1:** Summary Statistics of Genetic Diversity in *Plasmodium vivax*: Average Nucleotide Pairwise Differences (π), Nucleotide Diversity (θ), and Genetic Divergence (Jukes–Cantor corrected) between *P. vivax* and *Plasmodium cynomolgi* (*k*)

	π (95% CI)	θ (95% CI)	*k* (95% CI)
*P. vivax*	4.35 × 10^−3^ (3.59 × 10^−3^–5.11 × 10^−3^)	4.78 × 10^−3^ (3.94 × 10^−3^–5.62 × 10^−3^)	—
*P. vivax*/*P. cynomolgi*	—	—	0.145 (0.142–0.148)
*P. vivax*/*Plasmodium knowlesi*	—	—	0.183 (0.180–0.185)

Note.—CI, confidence interval.

**Table 2:** Summary of Fitting of the Generalized Linear Model of the MK Table to the *Plamodium vivax* Polymorphism and Divergence against *Plamodium cynomolgi*

Groups	Name	Variance	Standard Deviation	Corr		
Random Effects						
G	(Intercept)	1.72	1.31			
	R	0.158	0.398	0.037		
	F	1.695	1.302	−0.895	−0.178	
	RF	0.339	0.582	−0.097	0.351	−0.005
Fixed Effects						
	Estimate	Standard Error	*z* Value	Pr(>|z|)		
(Intercept)	−5.87	0.0241	−243.22	<2e-16***		
R	−0.99	0.0148	−66.79	<2e-16***		
F	4.34	0.0241	180.32	<2e-16***		
RF	−0.627	0.0165	−38.06	<2e-16***		

Note.—The model (as described in the Materials and Methods section is log_10_(counts) ∼ 1 + R + F + RF + (1 + R + F + RF|G). The Akaike information criterion for the fitting was 55,605. Total number of counts or mutations is 17,476 and 4,300 genes.

***P < 0.001.

The genes identified as being under positive selection are distributed all along the genome and are not clustered toward the regions with higher (or lower) diversity (fig. 4*c*). Among the genes identified by SnIPRE as being under positive selection, we again found genes that are potentially involved in immune response evasion (Kyes et al. 2007; Recker et al. 2011) such as RAD protein (Pv-fam-e, PVX_089805) and two Pf-fam-b proteins (PVX_101535 and PVX_002530).

## Discussion

All analyses aimed at understanding the distribution of variation genome wide and identifying signatures of selection rely on the identification of sets of orthologous genes between the compared genomes or ensuring that the assumption of synteny along the compared region is maintained. We found a similar number of orthologous genes between the three species to that reported by Tachibana et al. (2012), even after including genes that were present in at least one of the additional *P. vivax* genomes included in this study. However, we found a much larger number of annotated proteins unique to *P. vivax* (2,769 genes in this work vs. 204 in Tachibana et al. [2012]). The larger number of unique *P. vivax* genes is due to the fact that many of those loci were not found in all of the strains included. Our results seem to indicate two forces might contribute to the enrichment of genes uniquely present in *P. vivax*: 1) recent duplication events in genes within the *P. vivax* lineage and/or 2) misannotation of genes in *P. vivax* isolates that are uniquely found in a particular isolate. On one hand, we find a large number of paralogous genes shared between isolates of *P. vivax* that are not shared with the other species (*P. cynomolgi* and *P. knowlesi*), suggesting that recent duplications can explain the large number of unique genes present in *P.vivax*. This observation receives additional support whenever gene families are examined in detail, such is the case of the msp-3 gene family, which present recent duplications in *P. vivax* (Rice et al. 2014). On the other hand, the GO analysis and lack of assignment to known protein functions for the majority of unique genes found in each particular isolate of *P. vivax* suggests that such genes could be the result of misannotations (similar to what has been observed in plants, Bennetzen et al. 2004). Additional work on the annotated genes, particularly incorporating RNA-sequencing data to curate the proposed gene models in the newly sequenced genomes, will allow better characterization of the genes sets identified in *P. vivax* isolates and assess the importance of recent duplication events in the observed polymorphism.

As expected, we found a higher genetic diversity in *P. vivax* when compared with *P. falciparum,* which is the result of a combination of older time to most recent common ancestor (Jongwutiwes et al. 2005; Mu et al. 2005; Cornejo and Escalante 2006; Neafsey et al. 2012; Taylor et al. 2013) and potentially larger effective population sizes for *P. vivax* when compared with *P. falciparum* (Hughes and Verra 2001; Mu et al. 2005; Neafsey et al. 2012). The distribution of neutral polymorphism estimated from the number of segregating sites (θ_w_) and the average number of pairwise differences (π) are relatively similar. Nonetheless, we do observe a slight excess in the estimates of nucleotide diversity based on the number of segregating sites (θ_w_) with respect to average pairwise difference between sequences (see supplementary fig. S1, Supplementary Material online). This effect is also captured in the overall negative Tajima’s *D*, which could be the result of population expansions or negative selection genome wide, or both processes. Because of the small sample size (*n* = 5), we are extremely cautious about any strong conclusions regarding the demographics, yet the results are consistent with previous findings suggesting that *P. vivax* populations have experienced a population expansion (Mu et al. 2005, Taylor et al. 2013). Additional sequences of isolates will allow refining the inference of the demographic history in *P. vivax*.

The pattern of synonymous genetic diversity is not uniform along the genome of *P. vivax*, and it is noticeable that several chromosomes show higher nucleotide diversity in loci located in subtelomeric regions (fig. 3*a*), providing evidence that there is variation in the effective population size along the genome of *P. vivax.* This pattern has been observed in other species, including *Drosophila*, *Arabidopsis,* and humans (Gossmann et al. 2011). A similar pattern has been observed in *P. falciparum* in chromosome 2, in which nonvariable genes (now regarded as genes with low variability as more sequence data has accumulated) accounted for 82% of the genes close to the center of chromosome 2, whereas nearly 75% of the genes located in the subtelomeric regions were polymorphic (Kidgell et al. 2006). The regions close to the telomeres are also believed to recombine more frequently in *Plasmodium* (Figueiredo et al. 2002). These findings in malarial parasites are consistent with the observation in *Drosophila melanogaster* that the effective population size is positively correlated with recombination rates (Gossmann et al. 2011). In contrast, the observed pattern of neutral divergence between *P. vivax* and *P. cynomolgi* does not show an increase toward the ends of the chromosomes (fig. 3*b*), consistent with the prediction that divergence between these two species is only correlated with the mutation rates but not with the recombination rates (Begun and Aquadro 1992). Thus, these two observations of higher silent polymorphism within *P. vivax* toward the telomeres and uniform neutral divergence between *P. vivax* and *P. cynomolgi* are particularly important; they indicate changes in the effective population size along the *P. vivax* genome that could affect the efficiency of natural selection acting on genes located in those specific regions. Indeed, it has been proposed that the efficiency of positive and negative selection is reduced in areas of low recombination (Gossmann et al. 2011). Although no similar analyses have been done in other *Plasmodium*, these results complement previous observations indicating that many subtelomeric genes harbor species-specific gene families involved in host–pathogen interaction, as have been identified in *P. falciparum* and other *Plasmodium* species (Kooij et al. 2005). It follows that natural selection could be more efficient in the telomeric regions indicating that this particular genome architecture could be an overall adaptive mechanism that allows malarial parasites to cope with the changing environment such as the host immune system. Under this model, natural selection would not only affect and maintain the amount of genetic diversity in a given locus but also its relative position in the genome, favoring the maintenance of genes that could be contribute to adaptations under strong positive selection in areas with higher relative rates of recombination, such as the subtelomeric regions. Additional effort should be put toward understanding variation in gene composition, relative recombination rates, and the impact of selection on genes located in the subtelomeric regions. Indeed, a limitation in our study is that the methods used here do not account for how selection acts in these multigene families (e.g., vir and msp3), which may produce additional enrichment for signatures of positive selection in subtelomeric regions. Indeed, these genomes (Neafsey et al. 2012) were sequenced with a short read technology that makes the resolution of low complexity regions of the genome difficult. Such comparisons would require a far richer data set than the one available (Neafsey et al. 2012).

The observed enrichment of polymorphism in the subtelomeric regions is unlikely to be an artifact due to rearrangements and misalignment of reads to a reference genome. Indeed, all estimations of genetic polymorphism and divergence were performed on a locus-by-locus basis on a set of genes in which we have already proven orthology (1:1 relationship for all isolates and species considered), using the Salvador I genome as a reference (Carlton et al. 2008).

Most studies aimed to identify and understand signatures of selection rely on the assumption that the estimated synonymous polymorphism and divergence is neutral. Nonetheless, genes under strong translational selection present signatures of constraint in the evolution of synonymous sites due to strong purifying selection on the use of alternative codons (Ikemura 1985; Maside et al. 2004; Sharp et al. 2005), which can lead to false-positive results when testing for positive selection in other malarial parasites such as *P. falciparum* (Escalante et al. 1998, 2004). We found that *P. vivax* presents an even use of alternative codons genome wide, a pattern characterized by a large effective number of codons (supplementary fig. S4, Supplementary Material online). More importantly, the small fraction of genes that do present a higher codon usage bias (∼4% of genes with *N*_c_ values < 45) are not part of the set of genes for which we have identified signatures of selection. The lack of significant codon usage bias genome wide is in itself an interesting result given that strong bias is usually associated with pressure to increase translational efficiency and thus to produce proteins more rapidly. Indeed, most of the genes that present lower effective number of codons (*N*_c_) are involved in the expression machinery (i.e., RNA polymerase, ribosomal proteins, and translation initiation factors); we could speculate that protein expression itself seems to be under selection for translational efficiency in *P. vivax*. This is consistent with other observations indicating a significant correlation between frequency of specific codons in highly expressed genes and the abundance of tRNA (Rubio et al. 1996). The average codon usage bias measured by the same index (*N*_c_) is much stronger in *P. falciparum* than in *P. vivax*. However, it has been postulated that even when the effective number of codons alone suggests that *P. falciparum* is under stronger translational selection than *P. vivax*, most of the skewed codon usage can be attributed to compositional biases rather than stronger translational selection (Zilversmit et al. 2010). In fact, a stronger correlation between amino acid frequency in highly expressed genes and their corresponding tRNAs has been found in *P. vivax* than in *P. falciparum*, suggesting that translational selection has a more important role in *P. vivax*, even if the effect is weak or limited to a small fraction of genes (Yadav and Swati 2012).

The analyses performed with the modified MK test indicates that there is, overall, a very strong constraint (low proportion of nonlethal replacement polymorphism) and negative selection (highly conserved genes) in *P. vivax* (fig. 4*b*), with few genes identified as being under positive selection. These patterns contrast markedly with those observed in other eukaryotic organisms such as *Drosophila* that has numerous genes with signatures of positive selection even in the presence of strong constraint. These results could indicate a larger effective population size in *Drosophila* when compared with *P. vivax* (Smith and Eyre-Walker 2002; Bierne and Eyre-Walker 2004; Begun et al. 2007; Eilertson et al. 2012) suggesting that natural selection is more efficient in that species. Although this difference could be explained in part by the limited number of genomes included in this investigation, the patterns observed in *P. vivax* are more similar to those observed in humans, in which there are few genes with significant evidence of positive selection and a very strong signal of negative selection (Bustamante et al. 2005; Boyko et al. 2008; Eilertson et al. 2012). This suggests a small to moderate effective population size throughout the history of *P. vivax* that may correlate with the evolutionary history of human populations (Taylor et al. 2013). These results from the MK modified test are consistent with the observation that the effective population size of *P. vivax* (θ = 4.78 × 10^−^^3^, estimated in this work) is only about four times larger than the one estimated for humans (θ = 1 × 10^−^^3^) (Bustamante et al. 2005), and it is 100 times smaller than that the one estimated for *D. melanogaster* (θ = 0.01) (Begun et al. 2007; Karasov et al. 2010). Interestingly, *P. vivax* populations are clearly differentiated worldwide (Mu et al. 2005; Cornejo and Escalante 2006; Taylor et al. 2013), so additional genome sequences from diverse geographic locations will contribute to a better understanding of how this distribution of constraint and efficiency of selection changes among different geographic locations as a function of the changes in effective population size.

Our analyses illustrate how relevant the use of genome-wide polymorphism data is for the detection of patterns of selection, despite the small sample sizes per locus, having only five alleles per gene. Undoubtedly, the most interesting set of genes that show signatures of positive selection are those related to the “exportome” repertoire of *P. vivax* (Sargeant et al. 2006) and antigens like the putative MSP-7, genes for which there is increasing evidence of their expression during the infection (Sargeant et al. 2006; Westenberger et al. 2010), and for which it has been suggested a potential involvement in the escape of the parasite to the immune system. But equally interesting are a set of genes involved in processes that could have contributed to the adaptation of *P. vivax* to the human host but in a broader sense, for example, via finding new vectors rather than simply solving the problem of invading a new cell type, which has not been previously recognized as such.

The evidence of selection in other proteins involved in the control of the life cycle of the parasite suggests that there is more to be understood about the underlying genetic basis of adaptation of the pathogen to the human host, especially when it comes to understanding the basic biology of the life cycle of the parasite. *P. vivax* has marked differences in the timing for the development of different stages and the target cells, when compared with other *Plasmodium* species that infect humans. Such differences could have an effect on the efficacy of selection acting on different stages (Schneider and Escalante 2013). For instance, the helicases found under selection (specifically, the DEAD-BOX domain) are proteins putatively involved in the development of gametocytes (Mair et al. 2006) or that could be involved in other stage-specific processes. Although orthologs to some of these genes (such as DEAD-BOX helicase) have been recently highlighted as containing highly differentiated SNPs between isolates from regions with prevalent artemisinin resistance and susceptibility (Miotto et al. 2013; Takala-Harrison et al. 2013), it is unlikely that drug usage has been a selective pressure shaping variation on these genes in the examined isolates given that their collection predates artemisinin usage. We could speculate that these genes involved in gametocyte development could be selected by a variety of factors, including changes in the biology of the host that imposed restrictions in the timing and/or cellular context for the development of gametocytes.

Another interesting result is the finding that proteins involved in life cycle regulation such as a cyclin g-associated kinase (PVX_101265), potentially associated with changes of the parasite in the erythrocyte stages, show signatures of positive selection. Detailed analyses of the function of this protein and others showing signature of positive selection, like histone deacetylases, could help better understand their specific roles in the adaptation of the parasite to the human host and perhaps open the possibility to identify essential proteins that could serve as targets for treatment. Although intense efforts have been invested in understanding genes encoding antigens putatively under balancing selection to understand the parasite’ evasion of the host immunity (Escalante et al. 2004; Chenet et al. 2012; Krzyczmonik et al. 2012; Pacheco et al. 2012a), other genes presenting signatures of directional selection such as the putative small GTPase Rab1 could be key regulators of cytoskeleton dynamics and/or been involved in secretory activities (Jekely 2003). Unfortunately, there is no thorough understanding of this and other proteins functions in the *Plasmodium* cell.

The tests of selection employed in this study are intended to distinguish regions under directional selection, not balancing selection, a scenario for which our sample size is rather limited and severely underpowered. This means that we have identified genes that show more divergence and lower polymorphism than would be expected given the pattern observed in neutral genes along the *P. vivax* genome. We have not identified genes that show increased polymorphism in high frequency, which would be expected under a scenario of balancing selection, which is expected for antigens under selection by the host immune system. It is not surprising that proteins that have been identified or hypothesized as being under balancing selection like the apical membrane antigen 1, and circumsporozoite surface protein do not show up in our selection scans. Further analyses with increased sample sizes will allow surveying the genome for signatures of balancing selection and identify additional genes under a different form of positive selection.

## Supplementary Material

Supplementary tables S1–S4 and figures S1–S5 are available at *Genome Biology and Evolution* online (http://www.gbe.oxfordjournals.org/).

Supplementary Data
