# Supplementary material for: Genome-Wide Patterns of Genetic Polymorphism and Signatures of Selection in Plasmodium vivax
Source: Genome Biol Evol. 2014 Dec 17;7(1):106–19. doi: 10.1093/gbe/evu267 (PMC4316620; doi:10.1093/gbe/evu267)
Supplement: Supplementary Data [file supp_7_1_106__index.html]

Genome-wide patterns of genetic polymorphism and signatures of selection in Plasmodium vivax — Genome-Wide Patterns of Genetic Polymorphism and Signatures of Selection in Plasmodium vivax — Supplementary Data 

# Genome-Wide Patterns of Genetic Polymorphism and Signatures of Selection in *Plasmodium vivax*

## Supplementary Data

files

**Files in this Data Supplement:**

- Supplementary Data - pdf file
- Supplementary Data - pdf file
- Supplementary Data - pdf file
- Supplementary Data - pdf file
- Supplementary Data - pdf file
- Supplementary Data - docx file
