# Supplementary material for: Genome-Wide Patterns of Genetic Polymorphism and Signatures of Selection in Plasmodium vivax
Source: Genome Biol Evol. 2014 Dec 17;7(1):106–19. doi: 10.1093/gbe/evu267 (PMC4316620; doi:10.1093/gbe/evu267)
Supplement: Supplementary Data [file supp_evu267_Figures_S3.pdf]

Supplementary Figure S3. Null distributions for each regression coefficients corresponding to each of the10 knots of the B-spline design, as well as the observed fitted coefficient (black vertical lines) for each one of the 14 chromosomes in *Plasmodium vivax*. We consider significant only those observed fitted values that are above or below 99% of the regression coefficients form the bootstrapped pseudo-random samples.

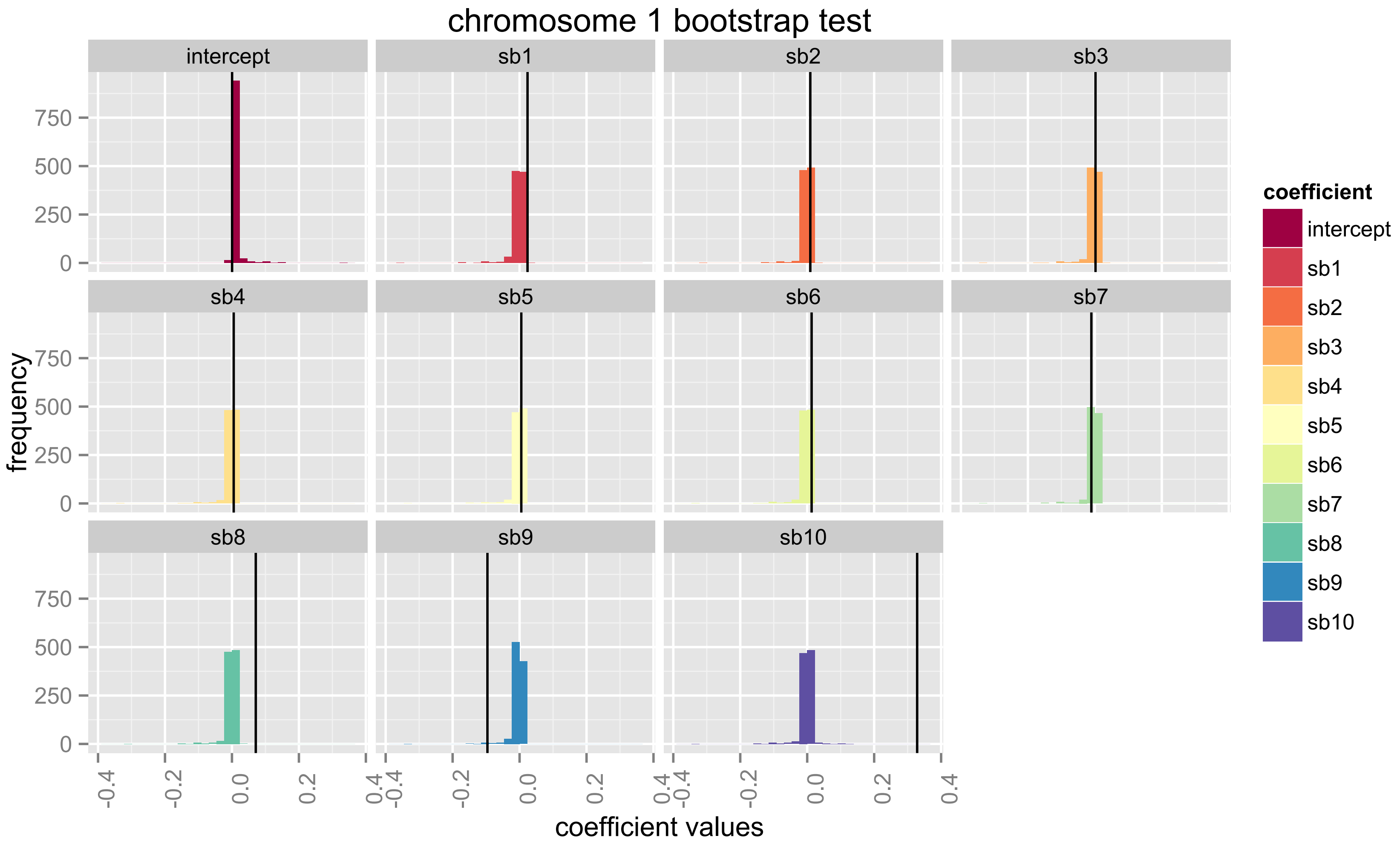

chromosome 1 bootstrap test
intercept
sb1
sb2
sb3
sb4
sb5
sb6
sb7
sb8
sb9
sb10
750
500
250
0
frequency
-0.4
-0.2
0.0
0.2
0.4
coefficient values
coefficient
intercept
sb1
sb2
sb3
sb4
sb5
sb6
sb7
sb8
sb9
sb10

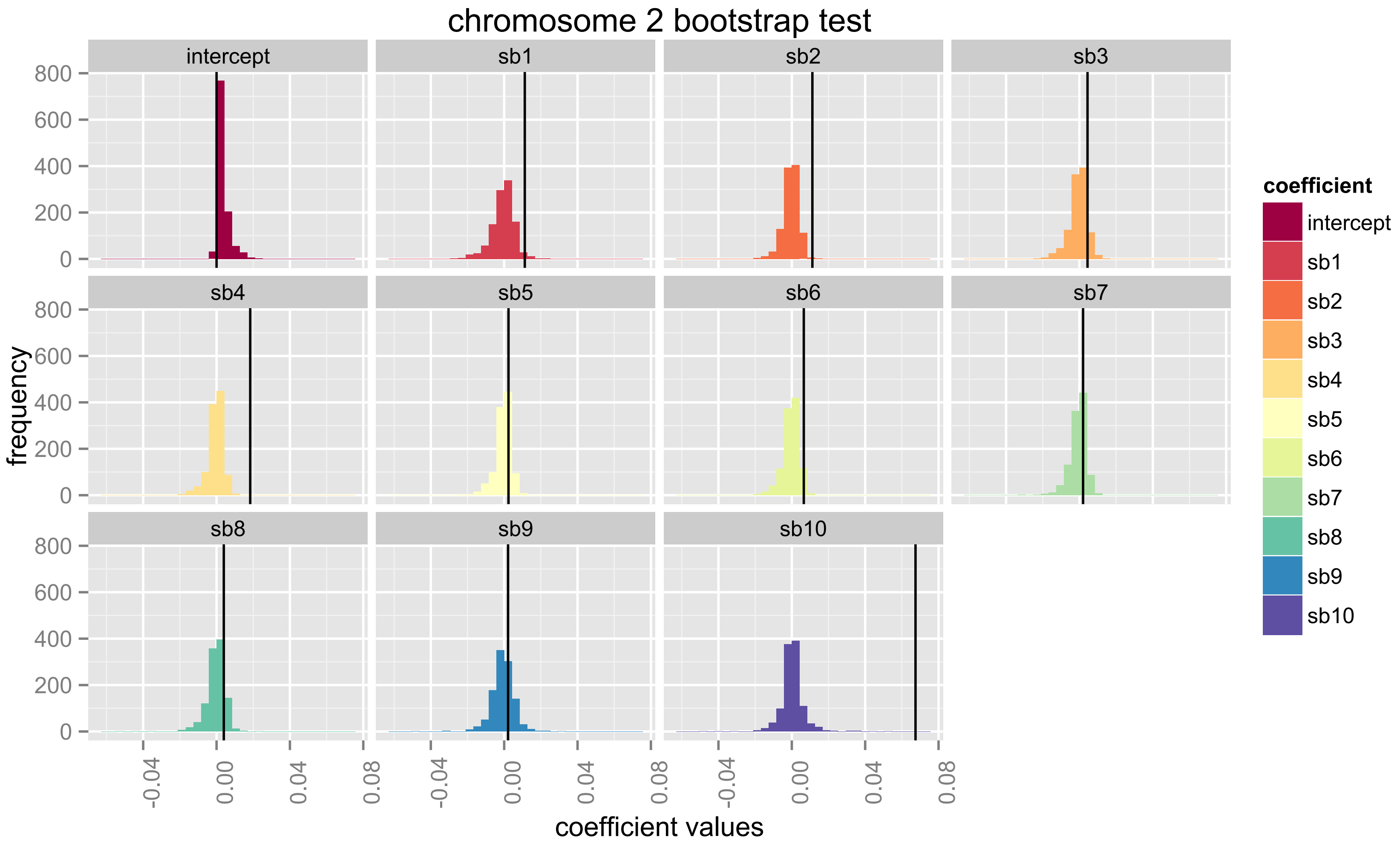
chromosome 2 bootstrap test
intercept
sb1
sb2
sb3
sb4
sb5
sb6
sb7
sb8
sb9
sb10
frequency
coefficient values
coefficient
800
600
400
200
0
-0.04
0.00
0.04
0.08

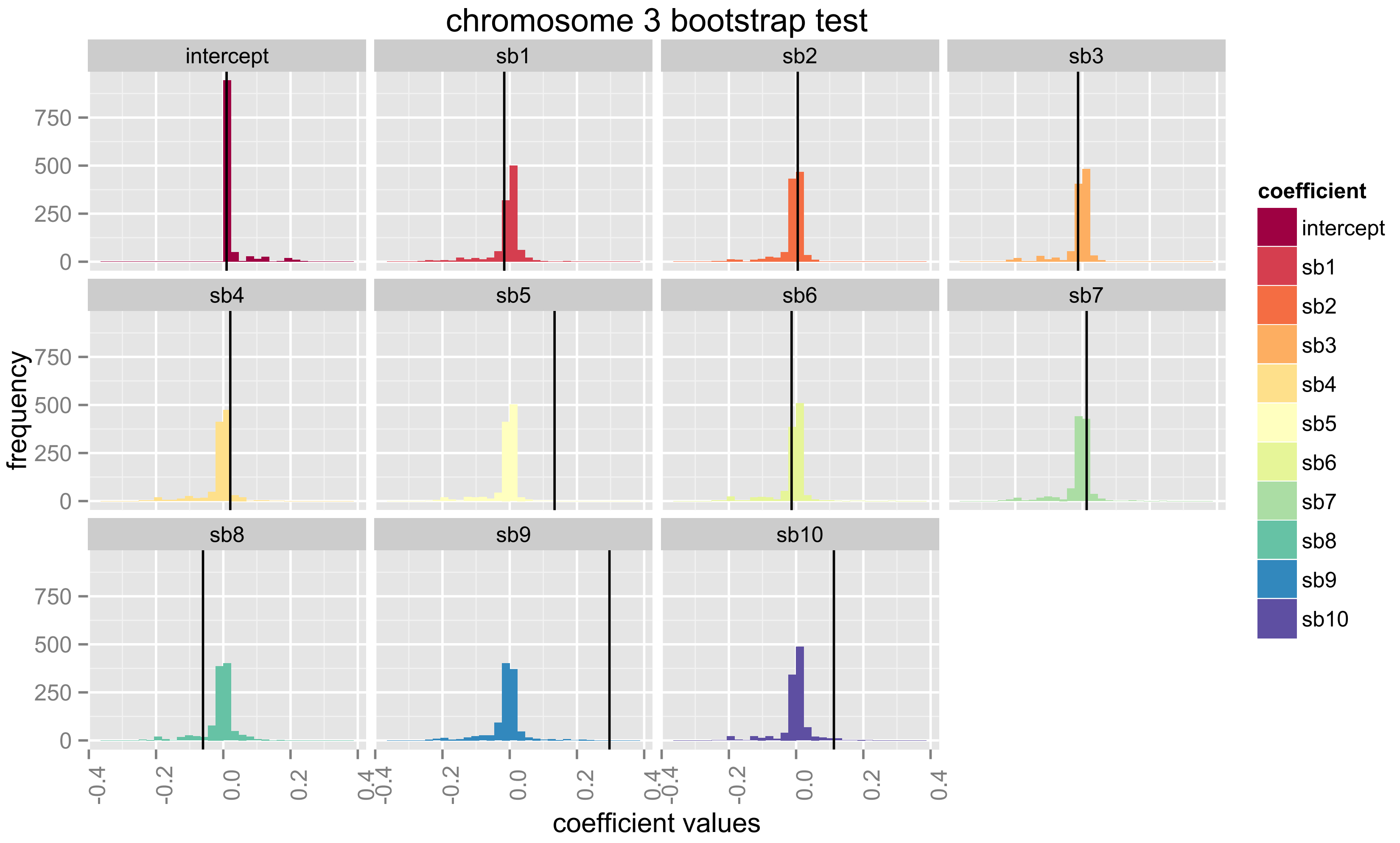

chromosome 3 bootstrap test
intercept
sb1
sb2
sb3
sb4
sb5
sb6
sb7
sb8
sb9
sb10
frequency
750
500
250
0
-0.4
-0.2
0.0
0.2
0.4
coefficient values
coefficient
intercept
sb1
sb2
sb3
sb4
sb5
sb6
sb7
sb8
sb9
sb10

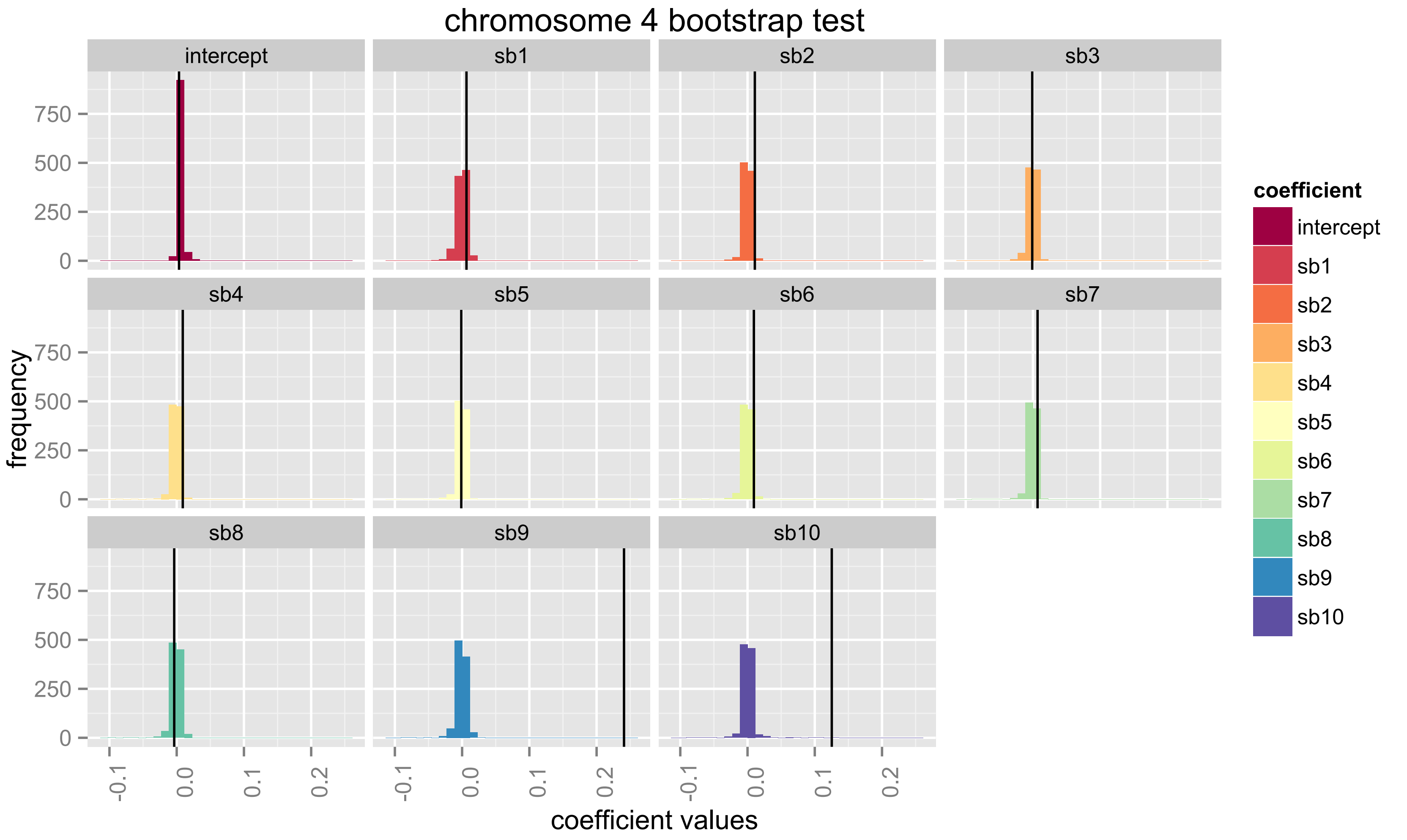
chromosome 4 bootstrap test
intercept
sb1
sb2
sb3
sb4
sb5
sb6
sb7
sb8
sb9
sb10
frequency
0
250
500
750
coefficient values
-0.1
0.0
0.1
0.2
coefficient
intercept
sb1
sb2
sb3
sb4
sb5
sb6
sb7
sb8
sb9
sb10

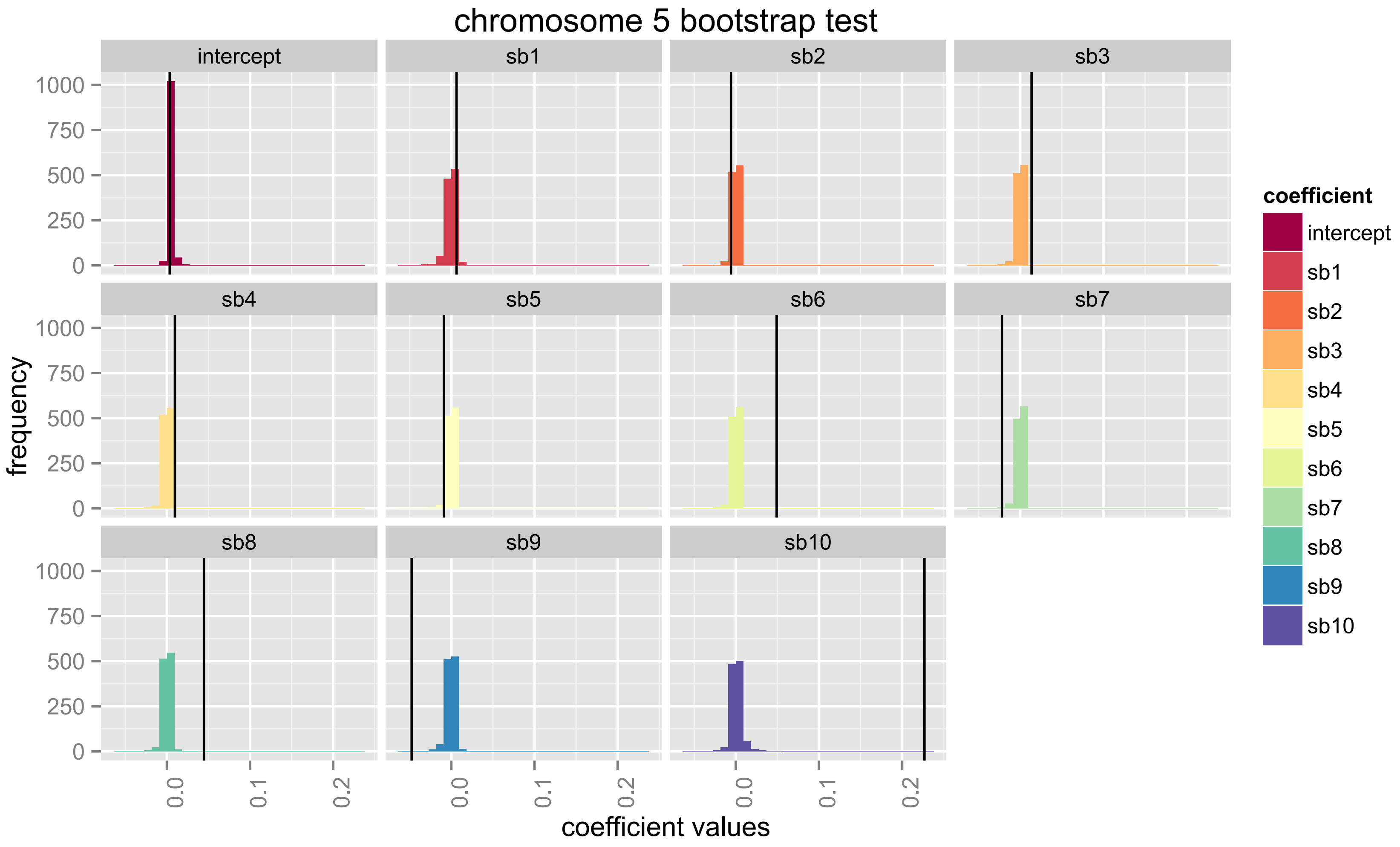

chromosome 5 bootstrap test
intercept
sb1
sb2
sb3
sb4
sb5
sb6
sb7
sb8
sb9
sb10
1000
750
500
250
0
frequency
0.0
0.1
0.2
coefficient values
coefficient
intercept
sb1
sb2
sb3
sb4
sb5
sb6
sb7
sb8
sb9
sb10

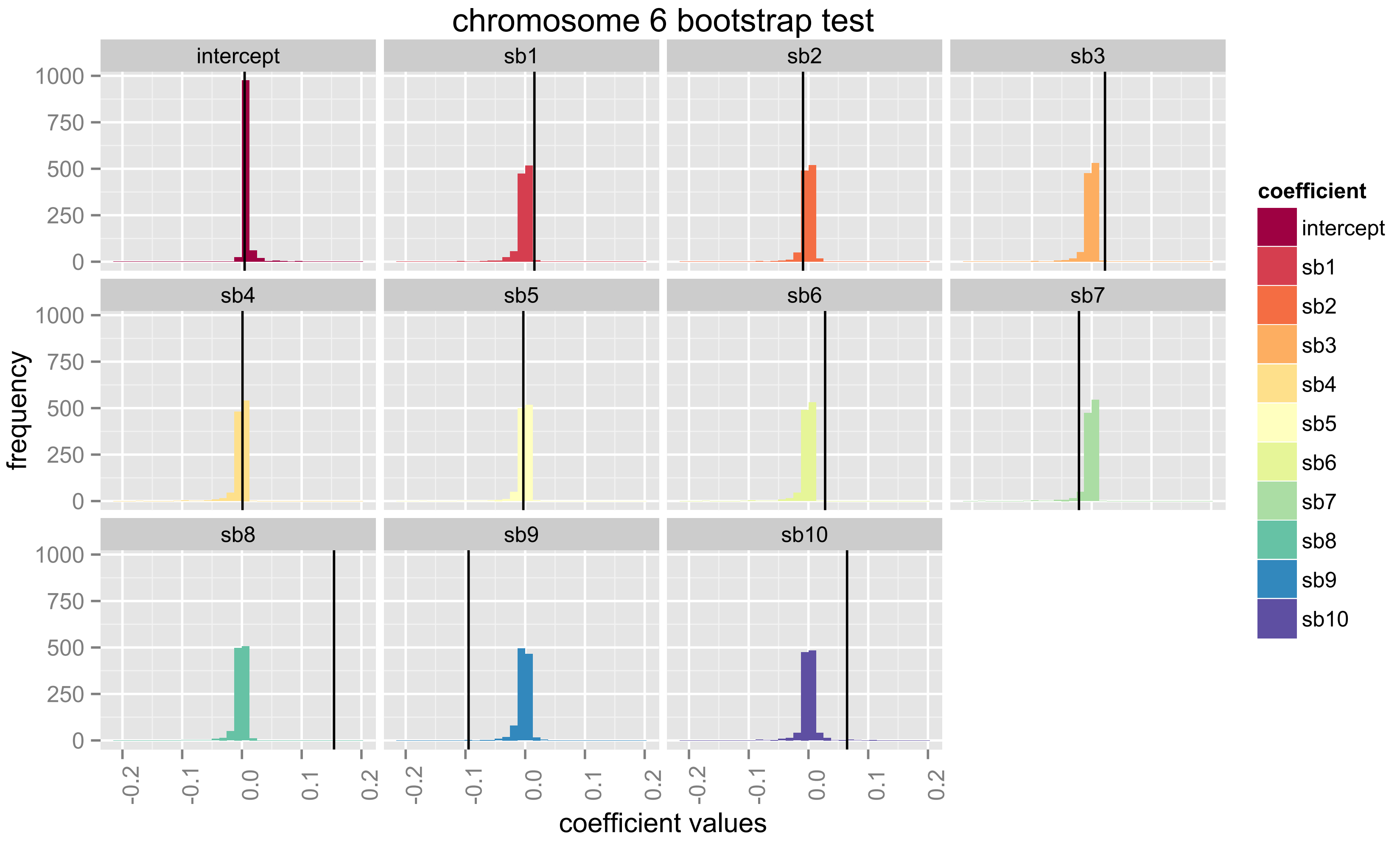
chromosome 6 bootstrap test
intercept
sb1
sb2
sb3
sb4
sb5
sb6
sb7
sb8
sb9
sb10
frequency
1000
750
500
250
0
-0.2
-0.1
0.0
0.1
0.2
coefficient values
coefficient
intercept
sb1
sb2
sb3
sb4
sb5
sb6
sb7
sb8
sb9
sb10

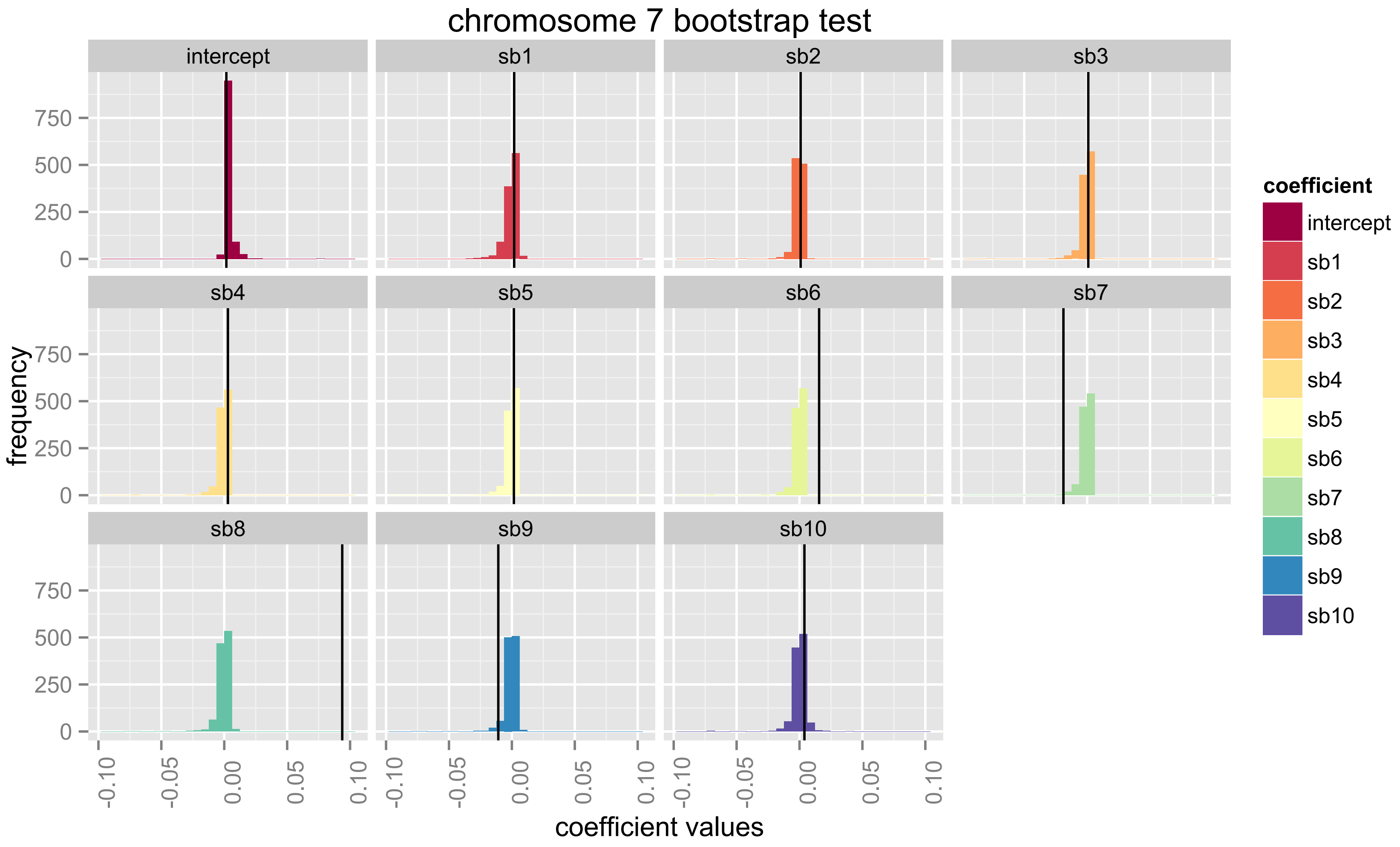

chromosome 7 bootstrap test
intercept
sb1
sb2
sb3
sb4
sb5
sb6
sb7
sb8
sb9
sb10
frequency
0
250
500
750
-0.10
-0.05
0.00
0.05
0.10
coefficient values
coefficient

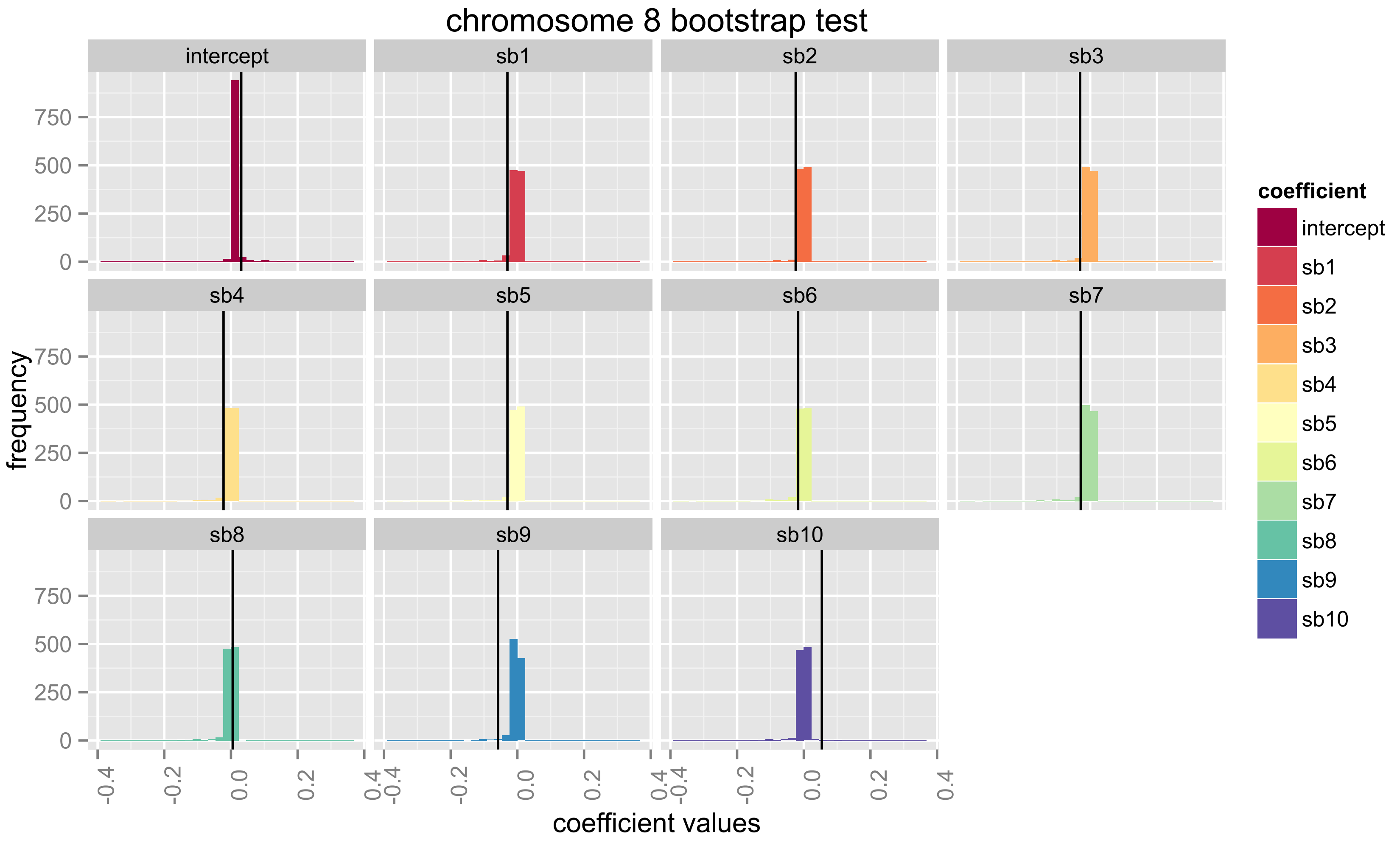
chromosome 8 bootstrap test
intercept
sb1
sb2
sb3
sb4
sb5
sb6
sb7
sb8
sb9
sb10
frequency
0
250
500
750
-0.4
-0.2
0.0
0.2
0.4
coefficient values
coefficient
intercept
sb1
sb2
sb3
sb4
sb5
sb6
sb7
sb8
sb9
sb10

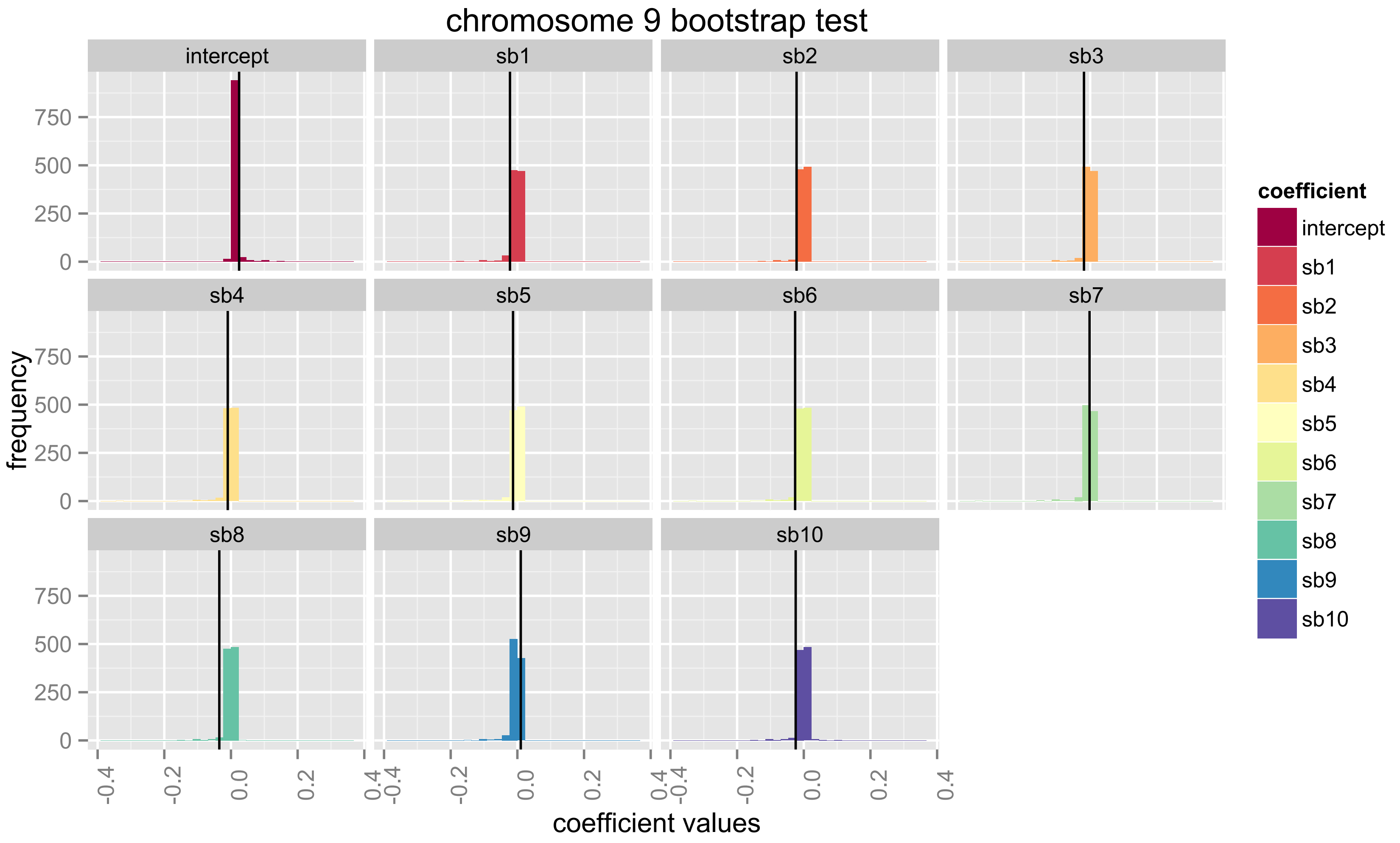

chromosome 9 bootstrap test
intercept
sb1
sb2
sb3
sb4
sb5
sb6
sb7
sb8
sb9
sb10
frequency
0
250
500
750
-0.4
-0.2
0.0
0.2
0.4
coefficient values
coefficient
intercept
sb1
sb2
sb3
sb4
sb5
sb6
sb7
sb8
sb9
sb10

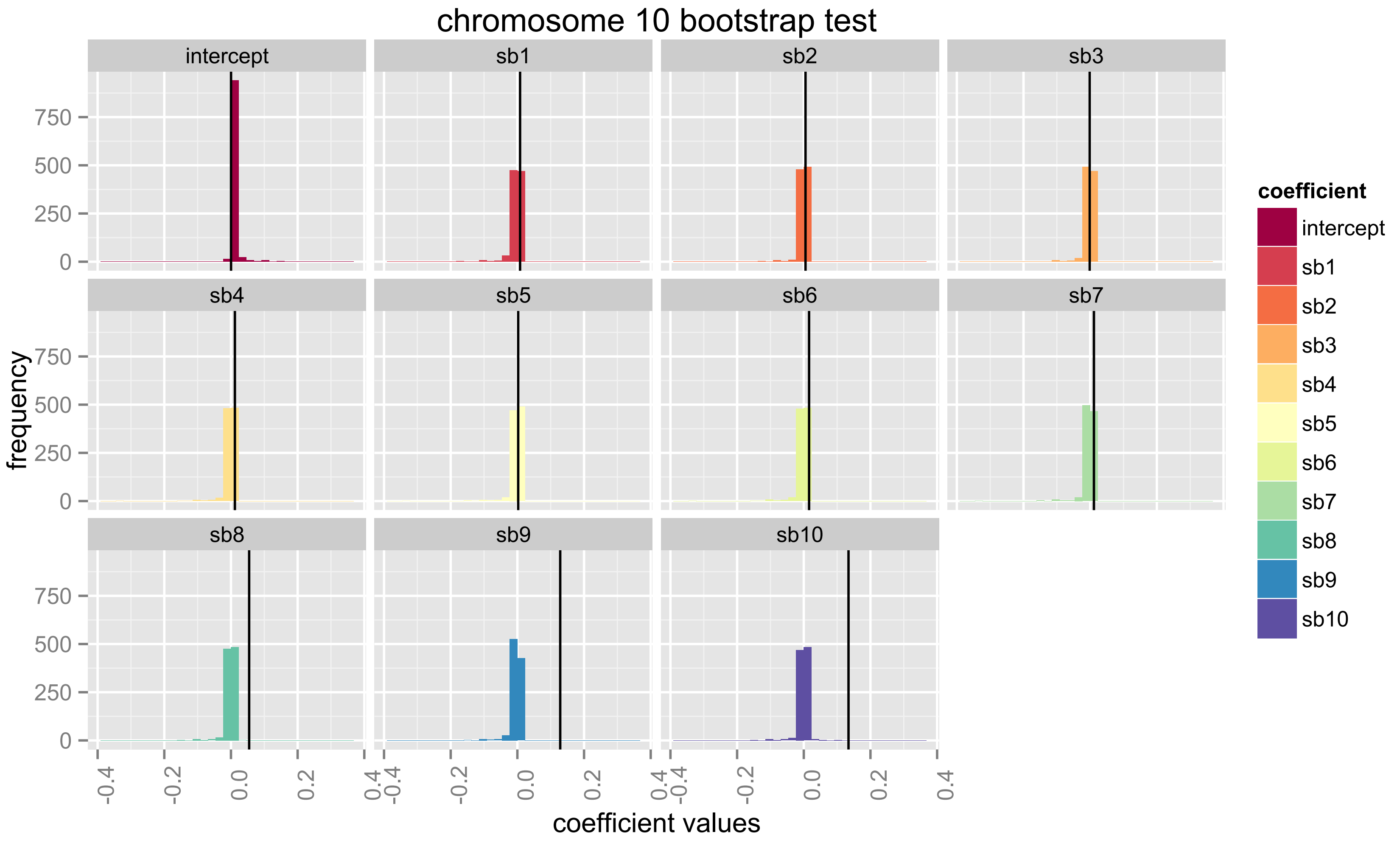
chromosome 10 bootstrap test
intercept
sb1
sb2
sb3
sb4
sb5
sb6
sb7
sb8
sb9
sb10
frequency
0
250
500
750
-0.4
-0.2
0.0
0.2
0.4
coefficient values
coefficient
intercept
sb1
sb2
sb3
sb4
sb5
sb6
sb7
sb8
sb9
sb10

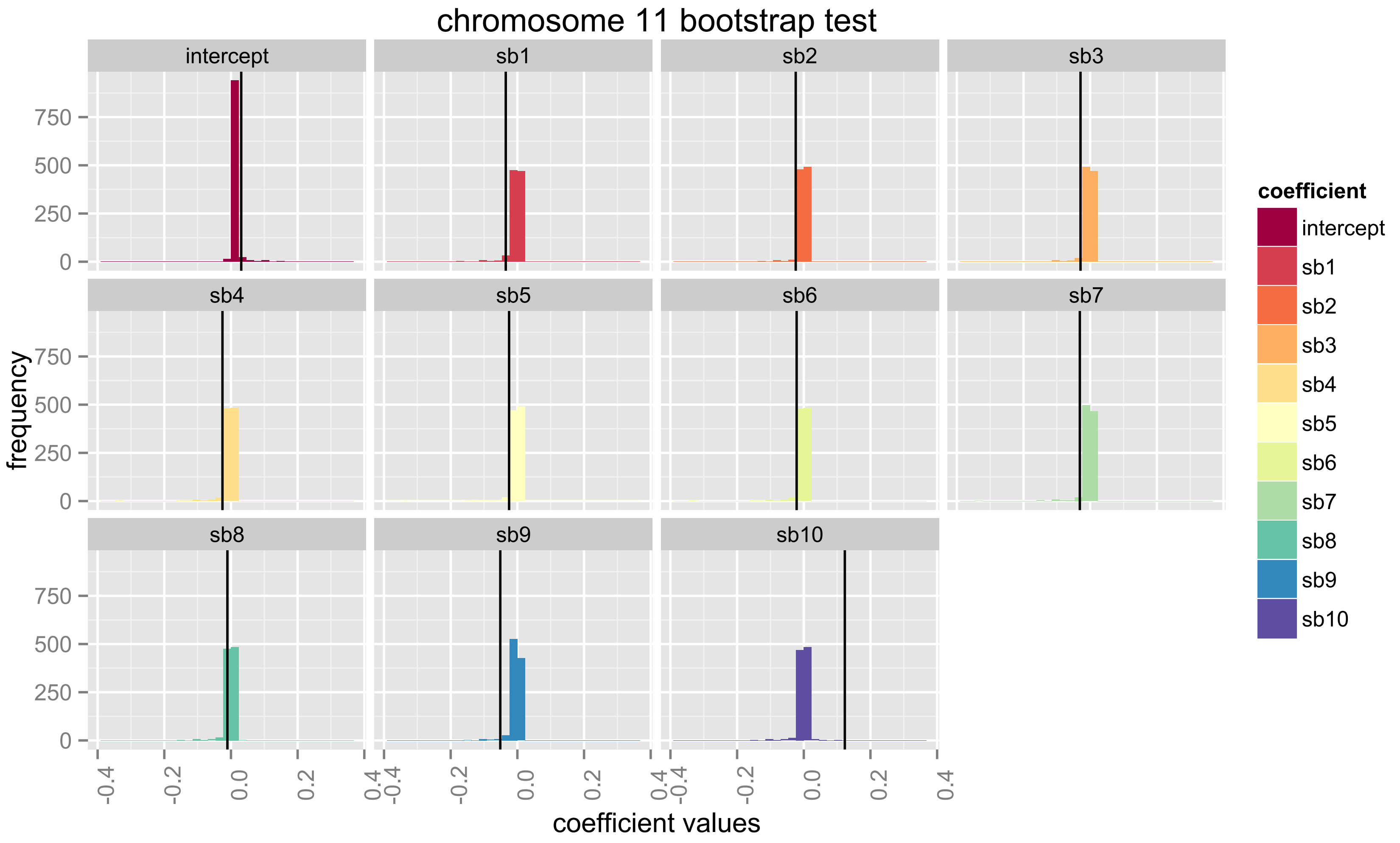
chromosome 11 bootstrap test
intercept
sb1
sb2
sb3
sb4
sb5
sb6
sb7
sb8
sb9
sb10
frequency
750
500
250
0
-0.4
-0.2
0.0
0.2
0.4
coefficient values
coefficient
intercept
sb1
sb2
sb3
sb4
sb5
sb6
sb7
sb8
sb9
sb10

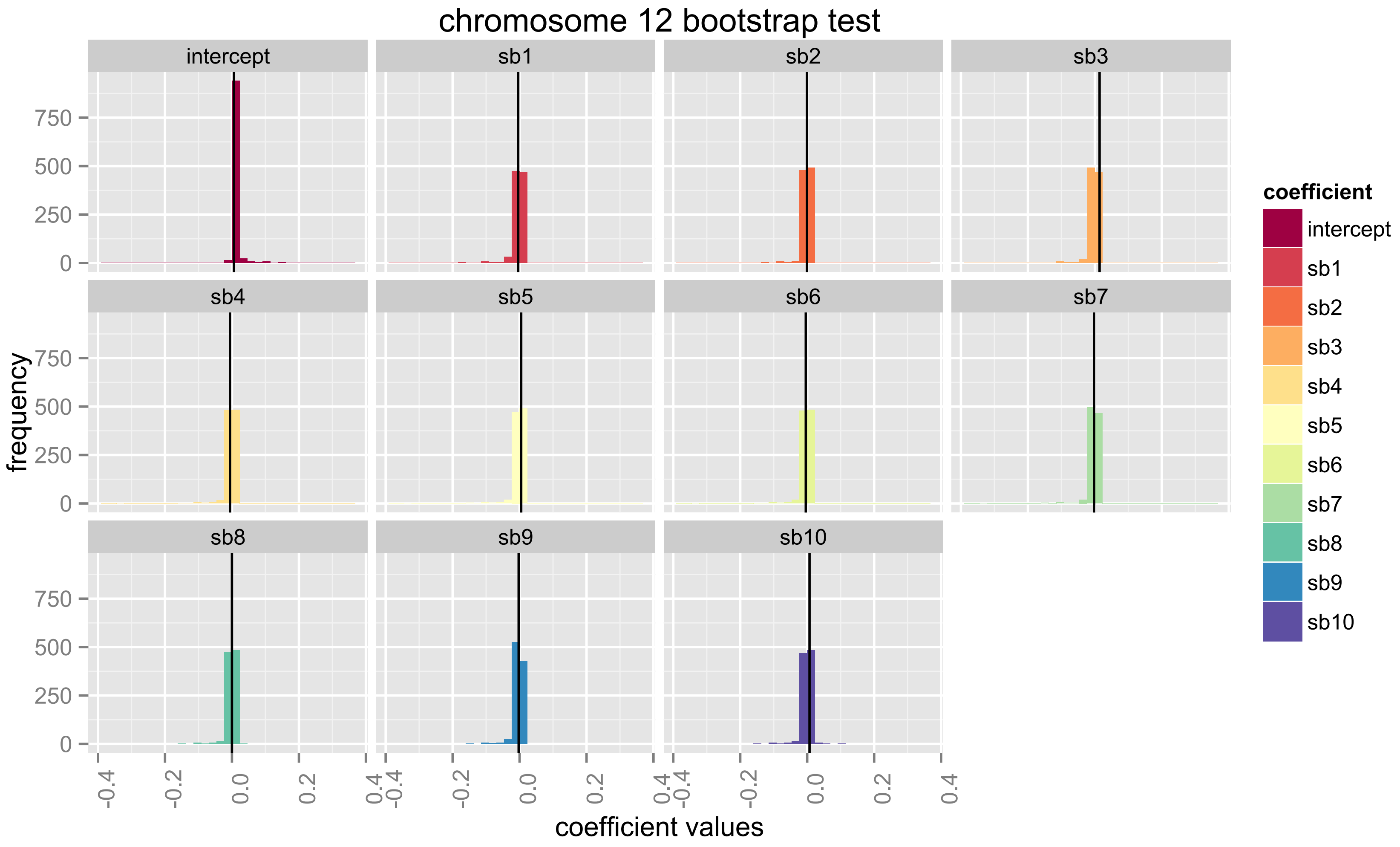
chromosome 12 bootstrap test
intercept
sb1
sb2
sb3
sb4
sb5
sb6
sb7
sb8
sb9
sb10
frequency
0
250
500
750
-0.4
-0.2
0.0
0.2
0.4
coefficient values
coefficient
intercept
sb1
sb2
sb3
sb4
sb5
sb6
sb7
sb8
sb9
sb10

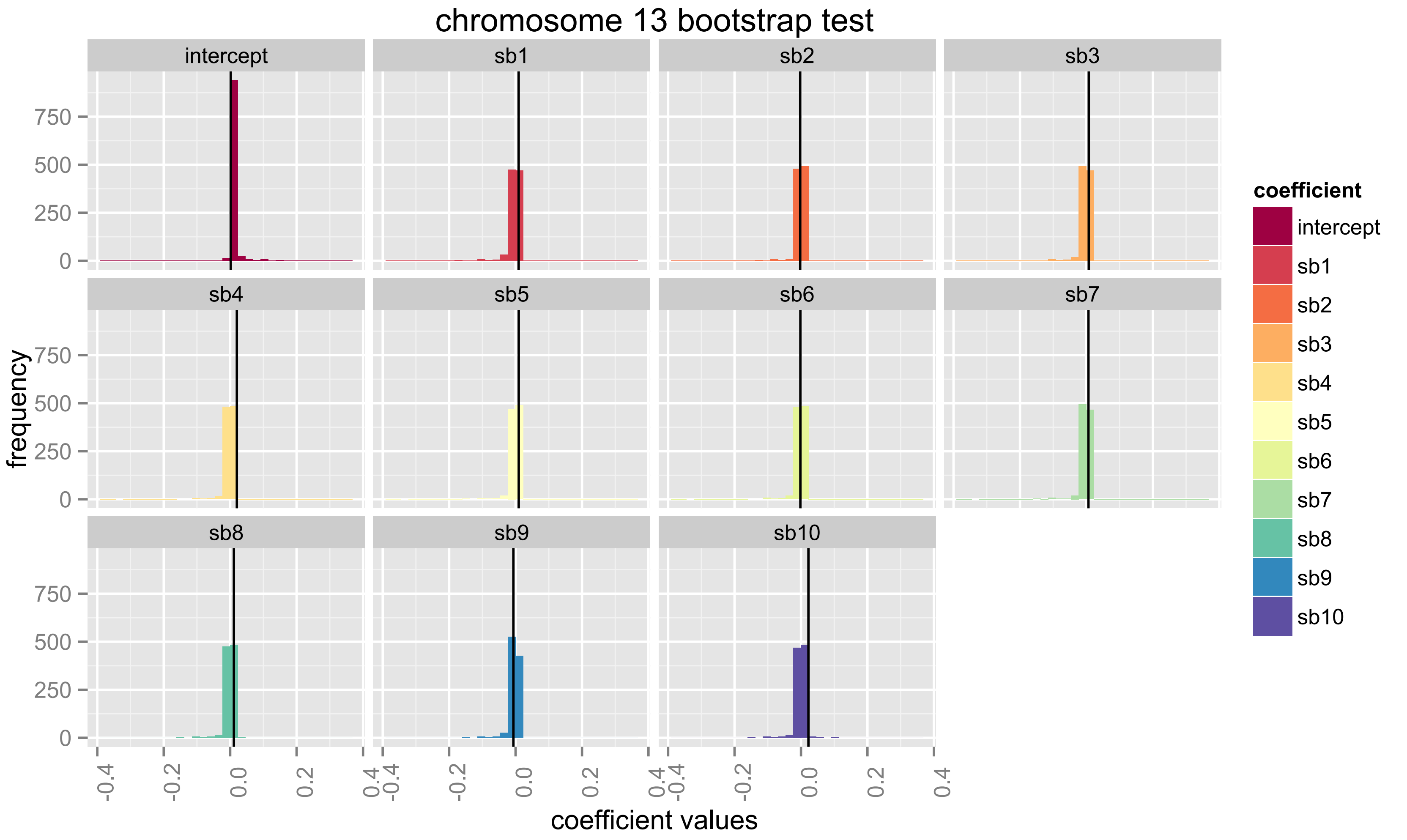
chromosome 13 bootstrap test
intercept
sb1
sb2
sb3
sb4
sb5
sb6
sb7
sb8
sb9
sb10
frequency
0
250
500
750
-0.4
-0.2
0.0
0.2
0.4
coefficient values
coefficient
intercept
sb1
sb2
sb3
sb4
sb5
sb6
sb7
sb8
sb9
sb10

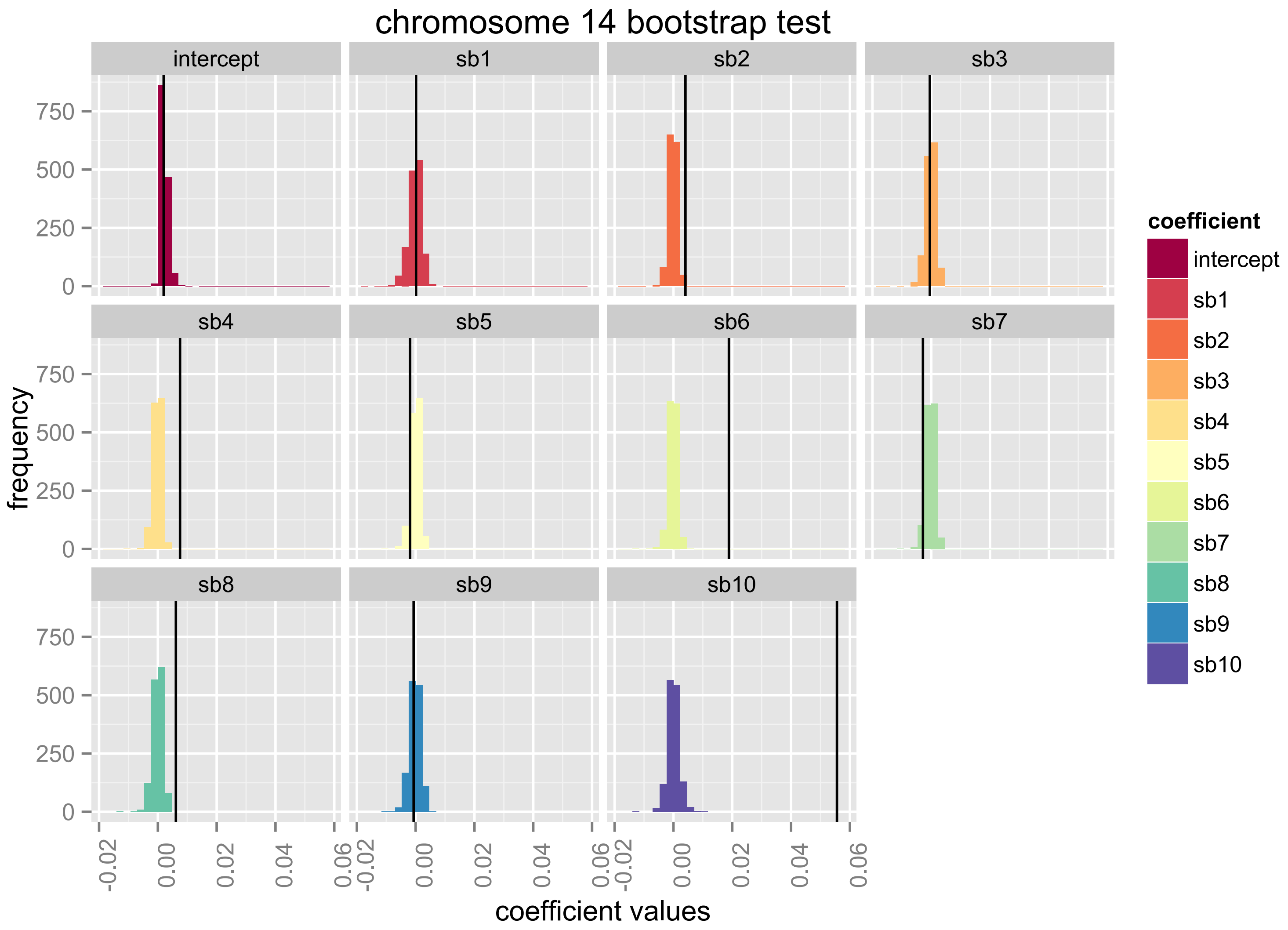
chromosome 14 bootstrap test
intercept
sb1
sb2
sb3
sb4
sb5
sb6
sb7
sb8
sb9
sb10
frequency
0
250
500
750
-0.02
0.00
0.02
0.04
0.06
coefficient values
coefficient
intercept
sb1
sb2
sb3
sb4
sb5
sb6
sb7
sb8
sb9
sb10
