# Supplementary figures and images for: Genome-Wide Patterns of Genetic Polymorphism and Signatures of Selection in Plasmodium vivax
Source: Genome Biol Evol. 2014 Dec 17;7(1):106–19. doi: 10.1093/gbe/evu267 (PMC4316620; doi:10.1093/gbe/evu267)

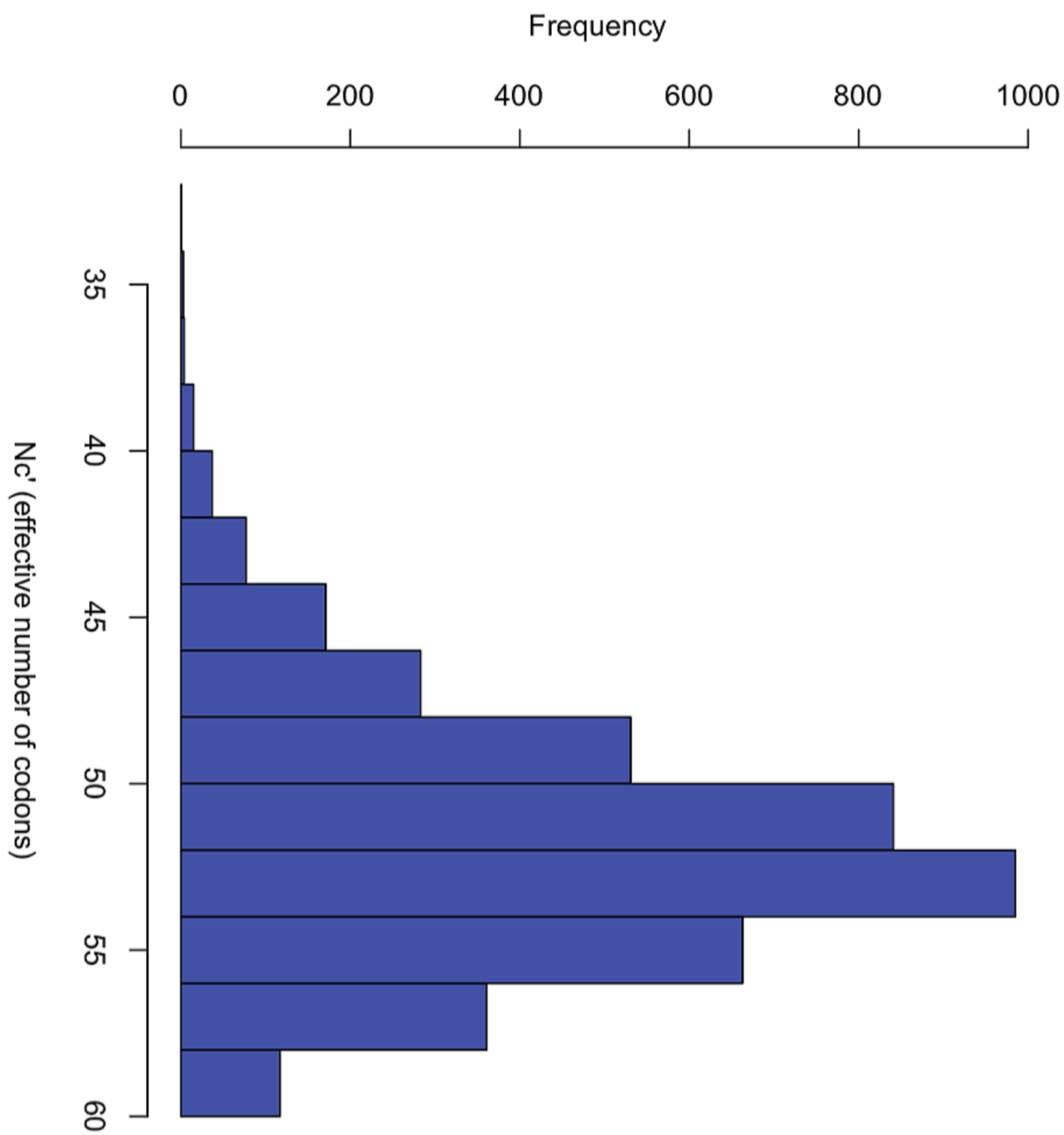

Supplement: Supplementary Data [file supp_evu267_Figures_S4.pdf]

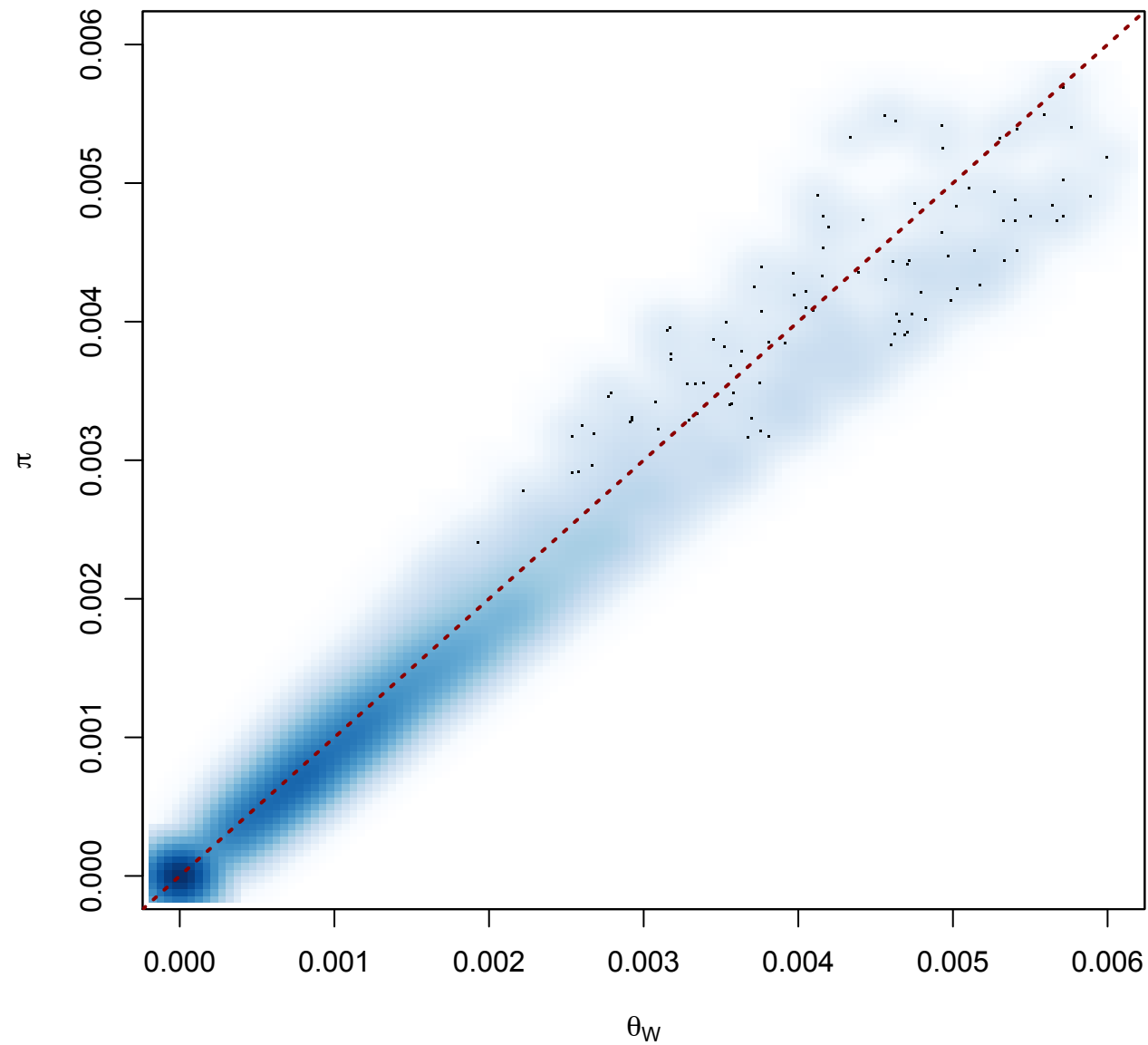

Supplement: Supplementary Data [file supp_evu267_Figure_S1.pdf]

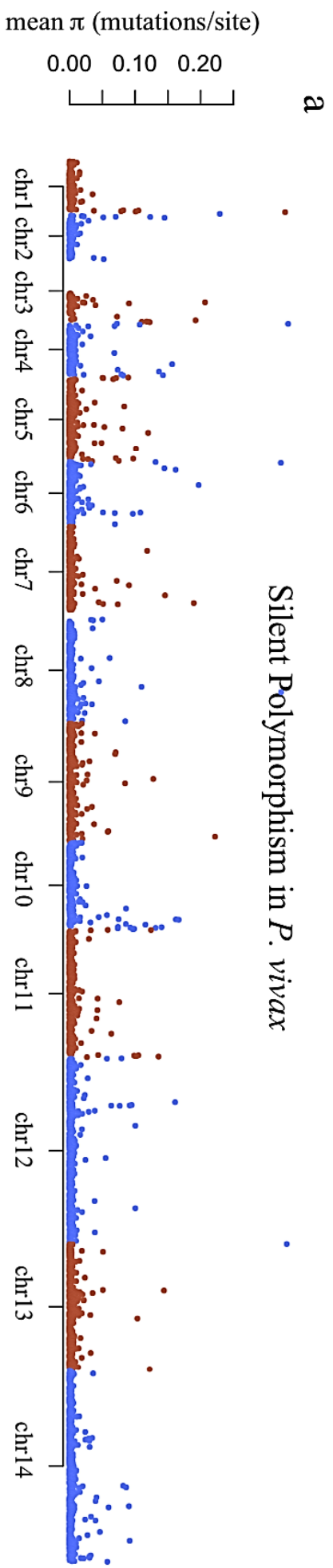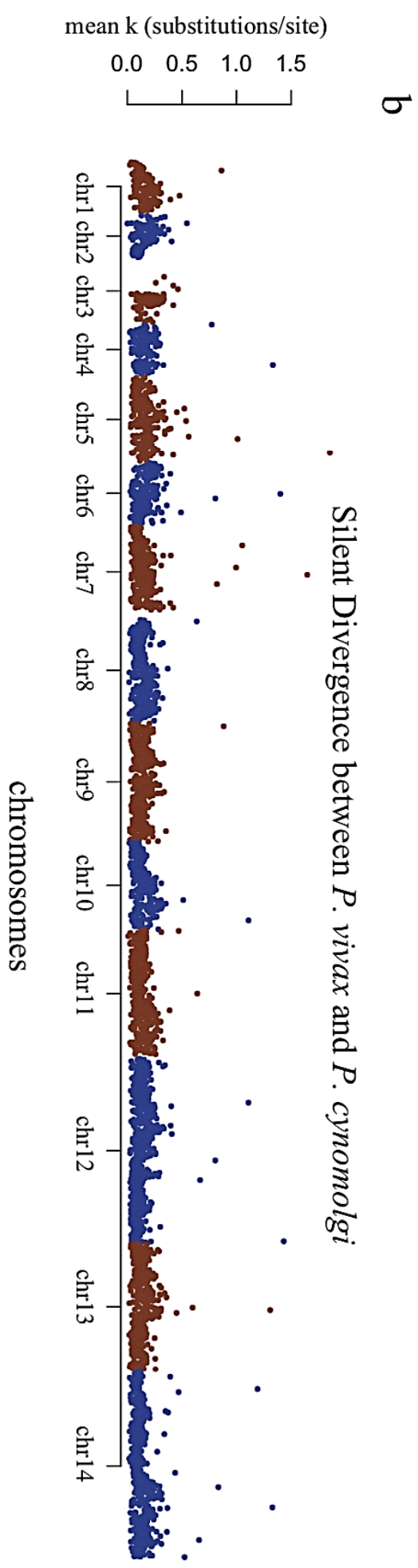

Supplement: Supplementary Data [file supp_evu267_Figure_S2.pdf]

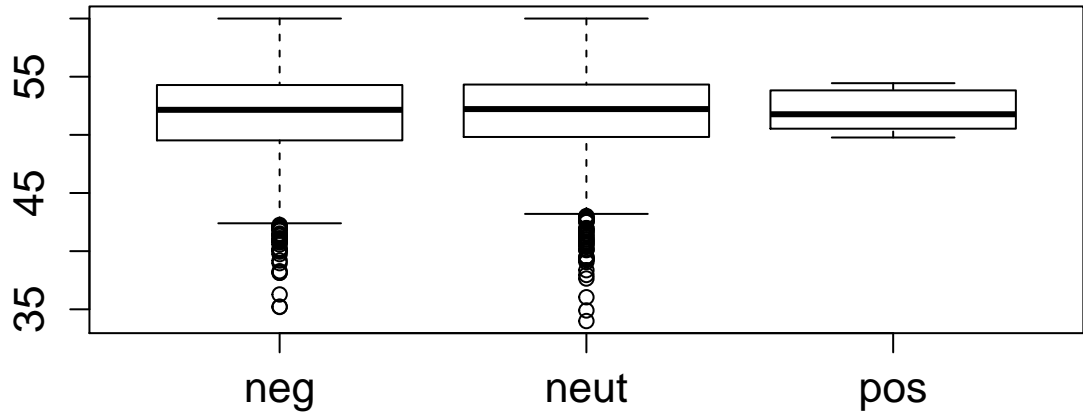

Supplement: Supplementary Data [file supp_evu267_Figure_S5.pdf]
