# Supplementary material for: Genome-Wide Patterns of Genetic Polymorphism and Signatures of Selection in Plasmodium vivax
Source: Genome Biol Evol. 2014 Dec 17;7(1):106–19. doi: 10.1093/gbe/evu267 (PMC4316620; doi:10.1093/gbe/evu267)
Supplement: Supplementary Data [file supp_evu267_GBE-Supplemental_Tables.docx]

Table S1. Set of reference genes for HKA test. Accession IDs are assigned using Salvador I as a reference.

| **gene ID** | **Gene product** |
| --- | --- |
| PVX_087950 | heat shock protein 86 putative |
| PVX_087990 | glutaredoxin domain containing protein |
| PVX_093650 | mannose-6-phosphate isomerase putative |
| PVX_081455 | calcium-transporting ATPase putative |
| PVX_000555 | calcium-dependent protein kinase 4 putative |
| PVX_096130 | 3-demethylubiquinone-9 3-methyltransferase putative |
| PVX_002665 | calcium-dependent protein kinase 1 putative |
| PVX_002930 | GDP-fucose transporter putative |
| PVX_003965 | transporter putative |
| PVX_003655 | aspartate aminotransferase mitochondrial precursor putative |
| PVX_088960 | protein disulfide isomerase putative |
| PVX_089165 | mitochondrial 2-oxoglutarate 2Fmalate carrier protein putative |
| PVX_089325 | 2-oxoglutarate dehydrogenase E1 component mitochondrial precursor putative |
| PVX_089950 | bifunctional dihydrofolate reductase-thymidylate synthase 1 putative |
| PVX_001740 | phosphomannomutase putative |
| PVX_111315 | protoporphyrinogen oxidase putative |
| PVX_110905 | pyruvate kinase putative |
| PVX_099230 | transporter putative |
| PVX_099530 | para-aminobenzoic acid synthetase putative |
| PVX_099535 | phosphoglycerate kinase putative |
| PVX_099600 | thioredoxin reductase 2 putative |
| PVX_099805 | fumarate hydratase putative |
| PVX_087090 | aspartyl aminopeptidase putative |
| PVX_086990 | vacuolar ATP synthase subunit E putative |
| PVX_094555 | Rho-GTPase-activating protein 1 putative |
| PVX_094635 | tubulin beta chain putative |
| PVX_094700 | hypothetical protein conserved |
| PVX_094815 | hypothetical protein conserved |
| PVX_094845 | phosphoglucomutase putative |
| PVX_095015 | enolase putative |
| PVX_095135 | cyclophilin putative |
| PVX_095405 | transporter putative |
| PVX_119610 | calcium-dependent protein kinase 3 putative |
| PVX_119565 | dual-specificity protein phosphatase putative |
| PVX_119350 | 1-cys-glutaredoxin-like protein-1 putative |
| PVX_091075 | hypothetical protein conserved |
| PVX_091100 | succinyl-CoA synthetase alpha subunit putative |
| PVX_091185 | actin putative |
| PVX_091465 | cathepsin C precursor putative |
| PVX_091545 | heat shock protein 90 putative |
| PVX_091650 | RNA splicing protein MRS2 mitochondrial precursor putative |
| PVX_092070 | hypothetical protein conserved |
| PVX_092245 | aquaglyceroporin putative |
| PVX_092310 | heat shock protein hsp70 homologue putative |
| PVX_092535 | Adenylate and Guanylate cyclase catalytic domain containing protein |
| PVX_092725 | coproporphyrinogen III oxidase putative |
| PVX_092880 | ABC transporter putative |
| PVX_080370 | f-actin capping protein beta subunit putative |
| PVX_098085 | topoisomerase I putative |
| PVX_097935 | subtilisin-like protease precursor putative |
| PVX_097770 | P-type ATPase putative |
| PVX_115205 | ferrochelatase putative |
| PVX_115190 | oxidoreductase aldo keto reductase domain containing protein |
| PVX_115140 | hypothetical protein 2C conserved |
| PVX_115030 | actin depolymerizing factor putative |
| PVX_114705 | vacuolar ATP synthase 21 kDa proteolipid subunit putative |
| PVX_114635 | hypothetical protein conserved |
| PVX_114575 | transmembrane amino acid transporter protein putative |
| PVX_114505 | Putative 6-pyruvoyl tetrahydrobiopterin synthase putative |
| PVX_114445 | pyruvate kinase putative |
| PVX_114430 | hypothetical protein conserved |
| PVX_114410 | oxidoreductase short-chain dehydrogenase family putative |
| PVX_114315 | hexokinase putative |
| PVX_114290 | superoxide dismutase putative |
| PVX_114265 | chorismate synthase |
| PVX_114230 | hypothetical protein 2C conserved |
| PVX_114050 | malate dehydrogenase putative |
| PVX_113615 | transporter protein putative |
| PVX_113540 | para-hydroxybenzoate--polyprenyltransf erase 284- hydroxybenzoate octaprenyltransferase 29 putative |
| PVX_083470 | glycerol kinase putative |
| PVX_083035 | phosphoenolpyruvate carboxykinase putative |
| PVX_083005 | aconitate hydratase I putative |
| PVX_082820 | calcium-dependent protein kinase putative |
| PVX_116630 | lactate dehydrogenase |
| PVX_116635 | dihydrofolate synthase 2Ffolylpolyglutamate synthase putative |
| PVX_116710 | vacuolar ATP synthase subunit G putative |
| PVX_117455 | hypothetical protein conserved |
| PVX_118170 | pyruvate dehydrogenase E1 beta subunit putative |
| PVX_118255 | fructose 1 6-bisphosphate aldolase putative |
| PVX_084225 | cytochrome c1 heme lyase putative |
| PVX_084315 | glyoxalase II putative |
| PVX_084580 | kinesin putative |
| PVX_084625 | P-type ATPase4 putative |
| PVX_084705 | cGMP-dependent protein kinase putative |
| PVX_084735 | glucose-6-phosphate isomerase putative |
| PVX_084965 | mitogen-activated protein kinase 1 putative |
| PVX_085435 | mannose-1-phosphate guanyltransferase putative |
| PVX_085490 | glutathione reductase putative |
| PVX_085995 | oxidoreductase aldo keto reductase domain containing protein |
| PVX_122015 | sodium 2Fhydrogen exchanger 1 putative |
| PVX_122425 | M1-family aminopeptidase putative |
| PVX_122485 | formyl transferase domain containing protein |
| PVX_122850 | Dihydrolipoyllysine-residue succinyltransferase component of 2-oxoglutarate dehydrogenase complex putative |
| PVX_123005 | hypothetical protein conserved |
| PVX_123080 | GDP-mannose 4 6-dehydratase putative |
| PVX_123230 | hydroxymethylpterin pyrophosphokinase-dihydropteroate synthetase putative |
| PVX_123745 | endoplasmin precursor putative |
| PVX_123830 | GTP cyclohydrolase I putative |
| PVX_123860 | cytochrome c heme lyase putative |
| PVX_124150 | hypothetical protein conserved |
| PVX_100555 | dihydrolipoamide dehydrogenase family protein |
| PVX_101195 | 5-aminolevulinic acid synthase putative |
| PVX_101280 | preprocathepsin c precursor putative |

Table S2. Set of genes with high relative codon usage bias (N_c_). Accession IDs are assigned using Salvador I as a reference

| **gene ID** | **Gene product** | **Nc** |
| --- | --- | --- |
| PVX_003555 | hypothetical protein, conserved | 44.1731 |
| PVX_115470 | Pv-fam-d protein | 44.3227 |
| PVX_086330 | exopolyphosphatase, putative | 41.9828 |
| PVX_086315 | proteosome subunit, putative | 43.8597 |
| PVX_090265 | tryptophan-rich antigen (Pv-fam-a) | 44.3228 |
| PVX_090255 | tryptophan-rich antigen (Pv-fam-a) | 40.7719 |
| PVX_090250 | tryptophan-rich antigen (Pv-fam-a) | 39.2526 |
| PVX_090195 | adapter-related protein complex 4 sigma 1 subunit, putative | 44.012 |
| PVX_086975 | cAMP-dependent protein kinase catalytic subunit, putative | 43.7148 |
| PVX_086985 | hypothetical protein, conserved | 42.9773 |
| PVX_099915 | RNA binding function, putative | 43.0228 |
| PVX_003705 | DNA-directed RNA polymerase II 16 kDa subunit, putative | 44.0503 |
| PVX_003720 | hypothetical protein, conserved | 44.0599 |
| PVX_092115 | 60S ribosomal protein L38-1, putative | 37.6456 |
| PVX_092065 | spermidine synthase, putative | 44.2014 |
| PVX_092040 | farnesyl pyrophosphate synthase, putative | 41.3098 |
| PVX_092025 | prefoldin subunit 5, putative | 44.423 |
| PVX_091965 | hypothetical protein, conserved | 43.0294 |
| PVX_098945 | hypothetical protein, conserved | 44.1751 |
| PVX_092765 | hypothetical protein, conserved | 43.2033 |
| PVX_003925 | hypothetical protein, conserved | 44.4268 |
| PVX_003955 | 60S ribosomal protein L37a, putative | 42.0389 |
| PVX_002930 | GDP-fucose transporter, putative | 43.0185 |
| PVX_002850 | hypothetical protein, conserved | 43.7244 |
| PVX_002675 | clathrin coat assembly protein AP17, putative | 36.0386 |
| PVX_002650 | 40S ribosomal protein S26, putative | 41.5148 |
| PVX_002570 | hypothetical protein | 35.2271 |
| PVX_119270 | exportin 1, putative | 43.7801 |
| PVX_119395 | hypothetical protein, conserved | 42.7463 |
| PVX_119435 | hypothetical protein, conserved | 40.4505 |
| PVX_119460 | hypothetical protein, conserved | 40.5643 |
| PVX_119480 | 60S ribosomal protein L7, putative | 40.9836 |
| PVX_119635 | eukaryotic translation initiation factor 3 subunit 11, putative | 39.7458 |
| PVX_119655 | hypothetical protein, conserved | 42.418 |
| PVX_119740 | 60S ribosomal protein L26, putative | 44.0394 |
| PVX_119780 | fusion protein, putative | 44.0584 |
| PVX_122380 | hypothetical protein, conserved | 38.9686 |
| PVX_122365 | hypothetical protein, conserved | 43.67 |
| PVX_122000 | vesicle-associated membrane protein 714, putative | 37.9535 |
| PVX_099815 | cytoplasmic dynein light chain, putative | 40.1165 |
| PVX_099780 | hypothetical protein, conserved | 43.8712 |
| PVX_099710 | hypothetical protein, conserved | 44.1557 |
| PVX_099650 | splicesome-associated protein, putative | 43.6085 |
| PVX_099585 | hypothetical protein, conserved | 43.8285 |
| PVX_099505 | hypothetical protein, conserved | 43.9668 |
| PVX_099450 | hypothetical protein, conserved | 44.3297 |
| PVX_099365 | nucleosome assembly protein 1, putative | 41.2614 |
| PVX_099245 | hypothetical protein, conserved | 41.7607 |
| PVX_099135 | phospholipid or glycerol acyltransferase, putative | 43.8636 |
| PVX_099020 | hypothetical protein, conserved | 40.801 |
| PVX_098990 | phosphatidylinositol N-acetylglucosaminyltransferase domain containing prot | 41.0672 |
| PVX_118585 | 50S ribosomal protein L20, putative | 40.9266 |
| PVX_118460 | hypothetical protein, conserved | 43.6433 |
| PVX_118130 | mitochondrial ribosomal protein S14 precursor, putative | 39.4952 |
| PVX_001675 | Phist protein (Pf-fam-b) | 43.7274 |
| PVX_001680 | Phist protein (Pf-fam-b) | 43.7906 |
| PVX_001910 | hypothetical protein, conserved | 39.3508 |
| PVX_001935 | hypothetical protein, conserved | 40.1882 |
| PVX_001970 | hypothetical protein, conserved | 34.9026 |
| PVX_111540 | hypothetical protein | 42.0554 |
| PVX_111325 | hypothetical protein, conserved | 43.7662 |
| PVX_111265 | WD domain, G-beta repeat domain containing protein | 44.4464 |
| PVX_111225 | transcription factor, putative | 43.8521 |
| PVX_081555 | hypothetical protein, conserved | 44.2783 |
| PVX_081335 | hypothetical protein, conserved | 43.0862 |
| PVX_081320 | elongation of very long chain fatty acids protein 3, putative | 42.6834 |
| PVX_115255 | ubiquitin/ribosomal, putative | 41.7833 |
| PVX_115310 | S-phase kinase-associated protein 1A, putative | 44.2377 |
| PVX_093660 | hypothetical protein, conserved | 43.9939 |
| PVX_093555 | proteasome beta-subunit type 4, putative | 42.6478 |
| PVX_088195 | hypothetical protein, conserved | 41.0266 |
| PVX_096280 | hypothetical protein, conserved | 41.9464 |
| PVX_096289 | hypothetical protein | 43.8197 |
| PVX_096300 | hypothetical protein, conserved | 43.5261 |
| PVX_096335 | 40S ribosomal protein S10, putative | 41.4122 |
| PVX_096340 | 60S ribosomal protein L11, putative | 42.3962 |
| PVX_096360 | serine/threonine-protein kinase NEK4, putative | 43.2507 |
| PVX_096365 | hypothetical protein | 43.7511 |
| PVX_095315 | hypothetical protein, conserved | 43.8762 |
| PVX_095270 | ubiquitin-conjugating enzyme E2, putative | 41.8227 |
| PVX_094785 | hypothetical protein, conserved | 39.0673 |
| PVX_094525 | thioredoxin domain containing protein | 43.8048 |
| PVX_089625 | eukaryotic translation initiation factor 3 subunit 4, putative | 43.2915 |
| PVX_089600 | hypothetical protein, conserved | 43.9047 |
| PVX_089530 | hypothetical protein, conserved | 42.6078 |
| PVX_089525 | hypothetical protein, conserved | 42.7393 |
| PVX_089310 | hypothetical protein, conserved | 43.3078 |
| PVX_089280 | 60S ribosomal protein L22, putative | 33.9912 |
| PVX_090935 | histone 2B | 43.371 |
| PVX_091140 | hypothetical protein, conserved | 42.6391 |
| PVX_091220 | hypothetical protein, conserved | 43.4622 |
| PVX_091495 | hypothetical protein, conserved | 35.2025 |
| PVX_091540 | clathrin assembly protein AP19, putative | 40.9191 |
| PVX_091670 | hypothetical protein, conserved | 39.193 |
| PVX_091815 | endoplasmic reticulum oxidoreductin, putative | 40.6638 |
| PVX_082455 | DnaJ domain containing protein | 43.8171 |
| PVX_082460 | ER lumen protein retaining receptor 1, putative | 43.6363 |
| PVX_082515 | tyrosine phosphatase, putative | 43.728 |
| PVX_082525 | 60S ribosomal protein L23, putative | 43.8804 |
| PVX_082720 | hypothetical protein, conserved | 41.4095 |
| PVX_082740 | hypothetical protein, conserved | 36.278 |
| PVX_083025 | sporozoite microneme protein, putative | 44.4499 |
| PVX_083045 | phosphoethanolamine N-methyltransferase, putative | 42.1346 |
| PVX_083330 | hypothetical protein, conserved | 44.4158 |
| PVX_083425 | hypothetical protein, conserved | 43.1926 |
| PVX_083525 | calcium-dependent protein kinase, putative | 43.5616 |
| PVX_097780 | hypothetical protein, conserved | 44.1555 |
| PVX_097805 | hypothetical protein, conserved | 42.8593 |
| PVX_097855 | hypothetical protein, conserved | 44.185 |
| PVX_097955 | hypothetical protein, conserved | 42.4317 |
| PVX_097970 | hypothetical protein, conserved | 40.5798 |
| PVX_098015 | soluble NSF attachment protein (SNAP), putative | 40.867 |
| PVX_080625 | CDK-activating kinase assembly factor, putative | 43.684 |
| PVX_080565 | hypothetical protein, conserved | 43.099 |
| PVX_080230 | U6 snRNA-associated Sm-like protein LSm4, putative | 39.5228 |
| PVX_079795 | hypothetical protein, conserved | 44.4881 |
| PVX_079760 | hypothetical protein, conserved | 43.8352 |
| PVX_117905 | hypothetical protein, conserved | 42.2575 |
| PVX_117835 | hypothetical protein, conserved | 43.7105 |
| PVX_117515 | hypothetical protein, conserved | 44.3103 |
| PVX_117410 | 30S ribosomal protein S10, putative | 42.6634 |
| PVX_117390 | 50S ribosomal protein S28e, putative | 38.3651 |
| PVX_117350 | hypothetical protein, conserved | 43.6957 |
| PVX_117315 | hypothetical protein, conserved | 43.7874 |
| PVX_117245 | hypothetical protein, conserved | 40.0899 |
| PVX_117040 | derlin-2, putative | 43.4541 |
| PVX_117030 | RNA helicase-1, putative | 44.4784 |
| PVX_116980 | hypothetical protein, conserved | 43.2933 |
| PVX_116960 | hypothetical protein, conserved | 44.2043 |
| PVX_116710 | vacuolar ATP synthase subunit G, putative | 42.8515 |
| PVX_116700 | 60S ribosomal protein L23a, putative | 38.2754 |
| PVX_116665 | hypothetical protein, conserved | 43.7562 |
| PVX_116600 | Tetratricopeptide repeat protein 11, putative | 44.1277 |
| PVX_113410 | hypothetical protein, conserved | 40.1798 |
| PVX_113440 | calcium-binding protein, putative | 41.1071 |
| PVX_113465 | long chain polyunsaturated fatty acid elongation enzyme, putative | 42.4979 |
| PVX_113535 | hypothetical protein, conserved | 40.7596 |
| PVX_113615 | transporter protein, putative | 43.998 |
| PVX_113860 | 60S ribosomal protein L19, putative | 38.1392 |
| PVX_113995 | alpha adaptin, putative | 43.8684 |
| PVX_114020 | histone H3, putative | 39.3616 |
| PVX_114055 | hypothetical protein, conserved | 43.2615 |
| PVX_114245 | hypothetical protein | 40.1146 |
| PVX_086235 | hypothetical protein, conserved | 42.6529 |
| PVX_086110 | hypothetical protein, conserved | 44.0729 |
| PVX_085895 | hypothetical protein, conserved | 38.1368 |
| PVX_085790 | Sperm-specific protein Don juan, putative | 42.0859 |
| PVX_085380 | hypothetical protein, conserved | 39.2884 |
| PVX_085355 | hypothetical protein, conserved | 43.4152 |
| PVX_085300 | calcium-dependent protein kinase, putative | 41.9752 |
| PVX_085225 | ribosomal protein L21e, putative | 42.0686 |
| PVX_085220 | basic transcription factor 3b, putative | 44.0619 |
| PVX_085175 | hypothetical protein, conserved | 42.5262 |
| PVX_084940 | hypothetical protein, conserved | 44.4642 |
| PVX_084910 | protein-L-isoaspartate O-methyltransferase beta-aspartate methyltransferase | 41.7048 |
| PVX_084515 | hypothetical protein, conserved | 40.0911 |
| PVX_084480 | hypothetical protein, conserved | 42.8913 |
| PVX_084400 | hypothetical protein, conserved | 42.5908 |
| PVX_084205 | hypothetical protein, conserved | 41.8077 |
| PVX_101360 | hypothetical protein, conserved | 40.8451 |
| PVX_101215 | myosin A tail domain interacting protein MTIP, putative | 41.5133 |
| PVX_101165 | 50S ribosomal protein L3, putative | 44.4525 |
| PVX_101160 | ubiquitin-conjugating enzyme E2, putative | 41.4313 |
| PVX_101075 | vacuolar sorting protein SNF7, putative | 39.959 |
| PVX_100855 | calcyclin binding protein, putative | 43.1707 |
| PVX_100665 | hypothetical protein, conserved | 43.7818 |
| PVX_100565 | hypothetical protein, conserved | 44.0266 |
| PVX_123960 | proliferating cell nuclear antigen, putative | 41.1554 |
| PVX_123935 | hydrolase, putative | 44.2088 |
| PVX_123920 | ubiquitin-activating enzyme e1, putative | 44.0439 |

Table S3. Genes under selection as identified by the modified HKA. Accession IDs are assigned using Salvador I as a reference. r/d refers to the ratio of polymorphism to divergence in each gene

| **gene ID** | **Gene product** |
| --- | --- |
| PVX_090245 | Phist protein (Pf-fam-b) |
| PVX_001695 | Phist protein (Pf-fam-b) |
| PVX_089805 | RAD protein (Pv-fam-e) |
| PVX_089840 | RAD protein (Pv-fam-e) |
| PVX_089460 | RAD protein (Pv-fam-e) |
| PVX_002530 | Pv-fam-b protein |
| PVX_084650 | DnaJ domain containing protein |
| PVX_082645 | merozoite surface protein 7 (MSP7), putative |
| PVX_117510 | DEAD-box RNA helicase, putative |
| PVX_111220 | RNA helicase, putative (development of gametocytes) |
| PVX_088190 | helicase, putative |
| PVX_099700 | histone deacetylase |
| PVX_099390 | sugar transporter, putative |
| PVX_099385 | protein disulfide isomerase, putative |
| PVX_081320 | elongation of very long chain fatty acids protein 3, putative |
| PVX_095300 | RNA-metabolising metallo-beta-lactamase domain containing protein |
| PVX_095120 | diacylglycerol O-acyltransferase, putative |
| PVX_089610 | dihydrolipoamide dehydrogenase, putative |
| PVX_091565 | histone acetyltransferase, putative |
| PVX_080540 | tubulin tyrosine ligase-like protein 1, putative |
| PVX_117110 | DNA-3-methyladenine glycosylase, putative |
| PVX_113465 | long chain polyunsaturated fatty acid elongation enzyme, putative |
| PVX_114465 | troponin c-like protein, putative |
| PVX_085265 | enoyl-CoA hydratase/isomerase family protein, putative |
| PVX_085055 | ribosomal protein L15, putative |
| PVX_080550 | small GTPase Rab1, putative |
| PVX_101265 | cyclin g-associated kinase, putative |
| PVX_087075 | 30S ribosomal protein S6, puatative |
| PVX_087085 | peptide release factor, putative |
| PVX_123935 | hydrolase, putative |
| PVX_123845 | polyadenylate-binding protein, putative |
| PVX_099655 | telomeric repeat binding factor 1, putative |
| PVX_002885 | Leu/Phe-tRNA protein transferase |
| PVX_119445 | FAD-dependent glycerol-3-phosphate dehydrogenase |
| PVX_119465 | T-complex protein 1, beta subunit, putative (the homologous in P. falciparum -PF3D7_0306800- is expressed in early and late gametocytes) |
| PVX_119545 | WD domain, G-beta repeat domain containing protein |
| PVX_097860 | 60S ribosomal subunit protein L24, putative |
| PVX_095125 | hypothetical protein, conserved |
| PVX_111565 | hypothetical protein, conserved |
| PVX_111535 | hypothetical protein, conserved |
| PVX_099190 | hypothetical protein, conserved |
| PVX_099425 | hypothetical protein, conserved |
| PVX_111360 | hypothetical protein, conserved |
| PVX_114730 | hypothetical protein, conserved |
| PVX_088280 | hypothetical protein, conserved |
| PVX_088110 | hypothetical protein, conserved |
| PVX_088105 | hypothetical protein, conserved |
| PVX_087780 | hypothetical protein, conserved |
| PVX_096260 | hypothetical protein, conserved |
| PVX_089535 | hypothetical protein, conserved |
| PVX_088950 | hypothetical protein, conserved |
| PVX_090960 | hypothetical protein, conserved |
| PVX_090985 | hypothetical protein, conserved |
| PVX_091450 | hypothetical protein, conserved |
| PVX_091645 | hypothetical protein, conserved |
| PVX_082430 | hypothetical protein, conserved |
| PVX_097900 | hypothetical protein, conserved |
| PVX_090100 | hypothetical protein, conserved |
| PVX_090025 | hypothetical protein, conserved |
| PVX_117125 | hypothetical protein, conserved |
| PVX_114025 | hypothetical protein, conserved |
| PVX_085040 | hypothetical protein, conserved |
| PVX_084875 | hypothetical protein, conserved |
| PVX_086945 | hypothetical protein, conserved |
| PVX_086980 | hypothetical protein, conserved |
| PVX_087050 | hypothetical protein, conserved |
| PVX_124165 | hypothetical protein, conserved |
| PVX_124105 | hypothetical protein, conserved |
| PVX_123925 | hypothetical protein, conserved |
| PVX_122615 | hypothetical protein, conserved |
| PVX_122585 | hypothetical protein, conserved |
| PVX_098940 | hypothetical protein, conserved |
| PVX_098610 | hypothetical protein, conserved |
| PVX_089545 | hypothetical protein |
| PVX_111510 | hypothetical protein |
| PVX_093685 | hypothetical protein |
| PVX_082425 | hypothetical protein |
| PVX_096015 | hypothetical protein |
| PVX_091995 | hypothetical protein |

Table S4. Genes under selection as identified by SnIPRE. Accession IDs are assigned using Salvador I as a reference.

| **gene ID** | **Gene Product** | **selection estimate (gamma)** |
| --- | --- | --- |
| PVX_089805 | RAD protein (Pv-fam-e) | 1.25 |
| PVX_101535 | Phist protein (Pf-fam-b) | 1.08 |
| PVX_002530 | Pv-fam-b protein | 0.92 |
| PVX_099330 | 50S ribosomal protein L3, putative | 0.88 |
| PVX_118682 | erythrocyte membrane protein 3, putative | 1.01 |
| PVX_090880 | phenylalanyl-tRNA synthetase beta chain, putative | 0.92 |
| PVX_097655 | 50S ribosomal subunit protein L28, putative | 1.11 |
| PVX_122145 | hypothetical protein, conserved | 1.26 |
| PVX_113225 | hypothetical protein | 1.15 |
| PVX_113240 | hypothetical protein | 1.30 |
| PVX_084095 | hypothetical protein | 1.46 |
| PVX_121940 | hypothetical protein | 0.99 |
| PVX_001655 | hypothetical protein | 1.52 |
